# Targeting Irgm1 to combat osteoporosis: suppressing ROS and restoring bone remodeling

**DOI:** 10.1038/s41419-025-07965-7

**Published:** 2025-08-27

**Authors:** Zichen Cui, Guanghui Gu, Fei Chen, Jianyi Li, Xiaofan Du, Shuqing Chen, Han Zhang, Chenxu Li, Jiale Shao, Jiayi Xi, Yukun Du, Qinghua Zhao, Yongming Xi

**Affiliations:** 1 Department of Orthopedics, The Affiliated Hospital of Qingdao University, Qingdao, China; 2 University of Illinois at Urbana-Champaign, Champaign, IL USA; 3 Department of Orthopedics, Shanghai General Hospital, Shanghai Jiao Tong University School of Medicine, Shanghai, China

**Keywords:** Diseases, Target validation

## Abstract

The accumulation of reactive oxygen species (ROS) leads to enhanced osteoclast activity, causing severe bone destruction in postmenopausal osteoporosis. Immunity-related GTPase family M member 1 (Irgm1) plays an essential role in affecting the production of intracellular ROS. To detect whether deletion of Irgm1 could suppress osteoclastogenesis through cellular redox regulation, we first evaluated whether the Irgm1 level was significantly elevated in mice bone marrow-derived monocytes/macrophages (BMDMs) from ovariectomy (OVX)-induced osteoporosis mice. Moreover, bioinformatics network analysis was performed to identify Irgm1 as a key upregulated gene during osteoclast differentiation. Next, we found that macrophage-specific Irgm1 knockout (Irgm1-cKO, Lyz2-Cre; Irgm1^flox/flox^) in OVX mice resulted in slower bone loss compared with OVX mice from the control group (Irgm1^flox/flox^). We then demonstrated that loss of Irgm1 inhibited osteoclast differentiation and bone resorption function via suppressing ROS accumulation. Further mechanism revealed that Irgm1 could endogenously bind to kelch-like ECH-associated protein 1 (Keap1) and keep Keap1 from ubiquitination and degradation. In the absence of Irgm1, Keap1 was downregulated and causing nuclear factor erythroid 2-related factor 2 (Nrf2) to translocate to the nucleus, thereby activating the level of the antioxidant system to combat oxidative stress. Moreover, Irgm1 deficiency in RAW264.7 promoted osteogenic differentiation of bone marrow mesenchymal stem cells (BMSCs) through inhibiting the M1 phenotype polarization. Taken together, our results revealed that loss of Irgm1 significantly alleviates OVX-induced bone loss, thus laying the foundation for exploring Irgm1 as a novel targeting approach for the treatment of osteoporosis.

## Introduction

The bone remodeling process maintains the integrity of bone structure under physiological conditions [1]. However, under pathological situations, including estrogen deficiency, hormone abuse, and aging, the enhanced bone resorption function caused by overactive osteoclasts relative to bone formation function breaks the balance of bone remodeling and results in osteoporosis [2]. Osteoporosis is a worldwide metabolic bone disease [3]. It is characterized by low bone mass, increased skeletal fragility, and fractures [4, 5]. As the aging population increases, osteoporosis has become a serious public health problem, causing a heavy economic burden [6]. Although drug treatments for osteoporosis have developed rapidly, their applications are limited due to side effects [7]. Therefore, it is urgent to fully elucidate the mechanism of osteoclast differentiation to further explore new drug therapies for osteoporosis.

Mature osteoclasts are the only cell type capable of bone resorption, and their overactivation is the main cause of postmenopausal osteoporosis [4]. With the lack of estrogen, the level of colony stimulating factor receptors and receptor activator of nuclear factor-κB (RANK) receptors on the surface of osteoclast precursor cells increases—their ligands are macrophage colony stimulating factor (M-CSF) and RANK ligand (RANKL), respectively, two key cytokines for osteoclastogenesis—making them easier to activate differentiation and maturation of osteoclast [8]. Reactive oxygen species (ROS), as second messengers in receptor-mediated signaling cascades, have been shown to be key factors in the differentiation and maturation of multinucleated osteoclasts [9]. ROS levels continuously increase during RANKL-induced osteoclastogenesis, which in turn further promotes the osteoclast differentiation and function. Excessive ROS upregulates TNF receptor-associated factor 6 (Traf6) expression, thereby activating the downstream nuclear factor κB (NF-κB) signal pathway and promoting nuclear translocation of P65. This cascade ultimately enhances the expression of nuclear factor of activated T-cells 1 (Nfatc1) [10]. Subsequently, osteoclast-specific markers, including tartrate-resistant alkaline phosphatase (TRAP) and cathepsin K (Ctsk) are upregulated to promote osteoclast maturation and bone resorption function [11]. Therefore, regulating excess ROS formation and thereby maintaining redox balance may be a potential approach to treat osteoclast-related diseases.

Nuclear factor erythroid 2-related factor 2 (Nrf2) is the most essential factor in maintaining cellular redox homeostasis [12, 13]. Under normal situations, Nrf2 combines with kelch-like ECH-associated protein 1 (Keap1), which restricts Nrf2 to the cytoplasm and inhibits Nrf2 activity [14]. However, as an adapter protein of Nrf2, the degradation of Keap1 seems to be accelerated under oxidative stress conditions, causing Nrf2 to be released from Keap1 and translocated into the nucleus, activating the level of an array of antioxidant genes, including Sod1, Sod2, Cat, Gpx, and Gr [12]. This suggests that the degradation process of Keap1 is regulated, but the factors involved in this regulation are still unclear. Accumulating evidence suggests that exposure of osteoclast precursor cells to excessive RANKL upregulates Keap1 and reduces Nrf2 nuclear translocation, leading to excessive accumulation of endogenous ROS and abnormal activation of osteoclasts [15, 16]. These studies indicate that there may be other proteins to crosstalk with the Keap1-Nrf2 axis during osteoporosis, thereby disrupting normal Keap1-Nrf2 signaling and maintaining Keap1 in a highly active state.

Immunity-related GTPase family M member 1 (Irgm1) was first discovered to be involved in interferon (IFN)-γ-regulated resistance to infection [17]. Lots of research has believed that Irgm1 plays a key role in a variety of diseases, including intracellular pathogen infection [18], autoimmune diseases [19], and stroke [20, 21]. A previous study demonstrated that Irgm1 controls actin ring polymerization in mouse macrophages and promotes their migration [20]. The F-actin rings are necessary for osteoclasts to perform bone resorption function [22]. However, the role of Irgm1 in osteoporosis is still unclear. Fang et al. demonstrated that Irgm1 regulates the production of ROS in macrophages, and scavenging ROS effectively inhibits Irgm1 overexpression-induced macrophage apoptosis [23]. In addition, Irgm1 mediates the macrophage polarization via the NF-κB signaling pathway, which is closely associated with osteoclast differentiation [24]. Nevertheless, Irgm1-mediated redox signaling whether this signaling pathway can restore bone loss caused by estrogen deficiency is still unclear.

Here, in the present study, we found that Irgm1 deficiency inhibited osteoclast differentiation and function, thereby effectively alleviating ovariectomy (OVX)-induced bone loss. More importantly, we found that Irgm1 protected Keap1 from degradation, and Irgm1 knockdown activated Nrf2 and reduced the over-accumulated ROS. These findings strongly suggested that Irgm1 showed a real effect on osteoclasts, a key factor in the process of menopausal osteoporosis, and provided a potential target for the prevention and treatment of osteoporosis.

## Materials and methods

### Reagents and antibodies

Cell culture reagents involving Dulbecco’s modified Eagle medium (DMEM; L110KJ; BasalMedia Technologies Co., Ltd), minimum essential medium α (α-MEM; L750KJ; BasalMedia Technologies Co., Ltd), and fetal bovine serum (FBS; S711-001S; Lonsera). We also used M-CSF (576406; Biolegend), RANKL (769404; Biolegend), N-Acetylcysteine (Nac; A9165; Sigma-Aldrich), cycloheximide (CHX; HY-12320; MCE), chloroquine (CQ; HY-17589A; MCE), MG132 (T2154; TargetMol, USA), 3-Methyladenine (3-MA; M129496; Aladdin), mifamurtide (HY13682; MCE), dexamethasone (D1756; Sigma-Aldrich), ascorbic acid (A92902; Sigma-Aldrich), and β-glycerophosphate disodium salt hydrate (G9422; Sigma-Aldrich) for osteoclasts and osteoblasts differentiation. Primary antibodies used for immunofluorescence and western blotting (WB) including anti-Irgm1 (71950; Cell Signaling Technology), anti-Runx2 (AF5185; Affinity), anti-Bmp2 (66383-1-IG; Proteintech), anti-Col1a1 (14695-1-AP; Proteintech), anti-Traf6 (WL04705; Wanleibio), anti-Nfatc1 (A1539; Abclonal), anti-Ctsk (DF6614; Affinity), anti-Nrf2 (16396-1-AP; Proteintech), anti-Keap1 (10503-2-AP; Proteintech), anti-Catalase (Cat; 21260-1-AP; Proteintech), anti-P65 (10745-1-AP; Proteintech), anti-p-P65 (3033; Cell Signaling Technology), anti-IκB (10268-1-AP; Proteintech), anti-p-IκB (2859; Cell Signaling Technology), anti-ubiquitin (Ub; 10201-2-AP; Proteintech), anti-IRGM (CQA6789; COHESION BIOSCIENCES), anti-Usp25 (12199-1-AP; Proteintech), anti-CD86 (13395-1-AP; Proteintech), anti-CD206 (18704-1-AP; Proteintech), and horseradish peroxidase (HRP)-GAPDH (CPA9165; COHENSION BIOSCIENCES). Secondary antibodies were obtained from Epizyme, including anti-mouse (LF101) and anti-rabbit (LF102).

### Animals

Conditional Irgm1 knockout mice model (Irgm1-cKO, Irgm1^flox/flox^; Lyz2-Cre) were screened by crossing Irgm1^flox/flox^ mice and Lyz2-Cre mice purchased from Cyagen Bioscience Company using a CRISPR/Cas9-mediated method. Irgm1^flox/flox^ mice were used as controls. The Qingdao University Laboratory Animal Management and Ethics Committee evaluated and authorized the animal experiment protocol (no. 202310C5708202502123). All mice were fed under a specific-pathogen-free condition in the Animal Laboratory Unit of Qingdao University, and all mice were provided with sterile food and water. Next, mice were divided randomly and subjected to bilateral OVX or sham-operation after anesthetizing with isoflurane at 2-month-old. Throughout the entire experimental process, investigators involved in data collection and outcome assessment remained blinded to group allocation. Any mouse showing severely affected vital signs was removed from the research. Three months after the surgery, all mice were sacrificed for further experiments. Polymerase chain reaction (PCR) genotyping primers are listed in Supplement Table 1.

Mice were treated with the Irgm1 inhibitor mifamurtide. Mifamurtide was injected into the tail vein at 1 mg/kg after OVX twice a week for 4 weeks.

### Micro-CT scanning

Left femurs fixed in 4% paraformaldehyde (PFA) from 5-month-old each group mice were collected were scanned by Micro-CT (μCT45; SCANCO). Quantitative tomography of the distal femoral metaphysis was performed, and bone volume fraction (BV/TV), trabecular number (Tb. N), trabecular thickness (Tb. Th), and trabecular spacing (Tb. Sp) were measured, and 3D images were reconstituted.

### Patients and sample collection

The clinical samples of human lamina tissues from osteoporosis and non-osteoporosis patients undergoing posterior lumbar laminectomy were procured following a process of obtaining informed consent from all patients, and the related investigations were approved by the Ethics Committee of the Affiliated Hospital of Qingdao University. All collected samples were immediately fixed in 4% PFA for subsequent experimental analyses.

### Bone histology analysis

The right femurs of mice from each group were fixed in 4% PFA and then decalcified in 10% EDTA at 4 °C for 3 weeks. The femurs were embedded in paraffin and sectioned into 7 μm-thick slices. Hematoxylin and eosin (H&E) staining, Masson staining, or TRAP staining were used to assess the histological changes.

Human tissues were isolated and fixed 4% PFA for 48 h. Then tissues were decalcified in 10% EDTA for 4 weeks. After embedding in paraffin, tissues were cut into 7 μm-thick tissue sections for immunohistochemical staining.

### Dynamic histomorphometry

To assess bone formation, we injected mice intraperitoneally with 25 mg/kg calcein on days 14 and 4 before sacrificing. After scanning for micro-CT, fixed left femurs were dehydrated in 20% sucrose for 12 h at 4 °C. Then we embedded the femurs in optimal cutting temperature (OCT) compound and cut with a microtome to 8 μm longitudinal sections by Cryofilm tape (Section Lab; Hiroshima). The sections were imaged under a fluorescence microscope [16].

### Cell isolation, culture, and differentiation

RAW264.7 and HEK293T cell lines were purchased from Wuhan Procell Life Science and Technology Co., Ltd. RAW264.7 and HEK293T were cultured in DMEM supplemented with 10% FBS in a humidified atmosphere at 37 °C and 5% CO_2_. RAW264.7 were seeded with 2 × 10^4^/ml and then cultured for 12 h. Next, α-MEM supplemented with 40 ng/ml RANKL and 10% FBS was replaced to induce osteoclasts.

BMDMs were flushed out from the tibias and femurs of 8-week-old control and Irgm1-cKO mice at 37 °C overnight. Adherent cells were incubated to the fourth generation as purified bone marrow mesenchymal stem cells (BMSCs) in α-MEM supplemented with 10% FBS. Non-adherent cells were collected. After adding the red blood cell lysate for 3 min, cells were seeded with 2 × 10^5^/ml in α-MEM with 10 ng/ml M-CSF and 10% FBS for 72 h. Then we incubated cells in α-MEM supplemented with 10 ng/ml M-CSF, 40 ng/ml RANKL, and 10% FBS for 5 days of osteoclast differentiation [11].

The fourth-generation BMSCs were seeded with 5 × 10^4^/ml. Osteoinduction medium (OIM; α-MEM supplemented with 50 μg/ml ascorbic acid, 10 mM β-glycerophosphate disodium salt hydrate, 100 nM dexamethasone, and 10% FBS) with different concentrations of conditioned medium was added after the cells reached 80% confluency. The media were replaced every two days. Cell culture materials such as culture bottles were purchased from Guangzhou Jet Bio-Filtration Co., Ltd.

### Cell proliferation assay

The proliferation of RAW264.7, BMDMs, and BMSCs was evaluated by Cell Counting Kit-8 (CCK-8; HY-K0301; MCE) assay. RAW264.7 cells with or without Irgm1 gene depletion were treated in α-MEM or DMEM with 10% FBS for 3 or 5 days. BMDMs were stimulated with different concentrations of mifamurtide (0-10000 nM) for 48 h. BMSCs were treated with OIM containing different percentages of conditioned medium from the indicated groups of RAW264.7 cells for 1, 3, or 5 days. Then, cells were incubated with 10 μL of CCK-8 solution at 37 °C for 2 h, followed by absorbance measurement at 450 nm using a spectrophotometer (SpectraMax Plus; Molecular Devices).

### TRAP staining and bone resorption assay

After 5 days of osteoclast differentiation, cells were fixed with 4% PFA for 15 min and stained by a tartrate-resistant alkaline phosphatase kit (387 A; Sigma-Aldrich). TRAP-positive cells with three or more nuclei were counted as osteoclasts under the bright field of a fluorescence microscope (Eclipse Ts2R; Nikon).

After osteoclasts matured in 48-well bovine cortical bone slices (2-0002-10; JoyTech), the medium was replaced with a 10% bleach solution to remove the cellular components. Then the slices were thoroughly rinsed with deionized water and air-dried. The absorption area was examined using a scanning electron microscope and evaluated using ImageJ software.

### F-actin staining

When the mature osteoclasts were formed, fixed with 4% PFA for 15 min. After washing cells with phosphate-buffered saline (PBS) three times, Phalloidin-iFluor (40736ES75; Yeasen) was used for staining F-actin rings at room temperature for 40 min in the dark. Cells were then incubated with 10 µg/ml 4′,6-diamidino-2-phenylindole (DAPI; C0065; Solarbio) at room temperature and visualized under a fluorescence microscope.

### Acridine orange (AO) staining

After osteoclasts reached maturation, cells were cultured with 10 μg/ml AO (A6014; Sigma-Aldrich) solution in α-MEM at 37 °C for 15 min. Then cells were washed with α-MEM three times, and the stained acid vesicles in osteoclasts were observed by a fluorescence microscope.

### ALP staining, ALP activity assay, and alizarin red staining

BMSCs were incubated with different treatments for 5 days. Following the fixation with 4% PFA, cells were treated with 5-bromo-4-chloro-3-indolyl phosphate (BCIP)/nitro blue tetrazolium ALP kit (C3206; Beyotime) for 30 min in the dark at 37 °C and observed using the bright field of a fluorescence microscope.

The ALP activity of each group was evaluated using an alkaline phosphatase activity assay kit (P0321M; Beyotime). After washing twice with PBS, the cells were lysed with RIPA lysis buffer without phosphatase inhibitor. Then, the supernatant was mixed with the working solution containing para-nitrophenyl phosphate (pNPP) substrate and incubated for 10 min at 37 °C. After the addition of a stop buffer, the absorbance at 405 nm was detected using a spectrophotometer.

After incubation with different treatments for 21 days, fixed BMSCs were washed three times with PBS and stained using alizarin red S solution (1%, pH 4.2) (G1452; Solarbio) for 30 min. Calcium nodules stained by alizarin were observed using a microscope after washing with double-distilled water.

### WB and Co-immunoprecipitation (Co-IP)

WB was conducted as described previously [11]. Briefly, the whole cell proteins were lysed from samples by cold radioimmunoprecipitation assay buffer (R0020; Solarbio) with phosphatase inhibitors (CW2383; Cwbio) and protease inhibitor (CW2200; Cwbio). After quantifying the concentration of proteins by a BCA assay kit (PC0020; Solarbio), 30 μg protein samples were separated on 10% SDS-PAGE gels by electrophoresis and transferred onto a 0.22 μm PVDF membrane (IPVH00010; Millipore). After blocking with 5% not-fat milk for 1 h at room temperature, the membranes were incubated with the primary antibodies at 4 °C overnight. After incubating with the respective secondary antibodies at room temperature for 1 h, the membranes were investigated by a super ECL detection reagent (36208ES60; Yeasen). Quantitative analysis of band densities was conducted using ImageJ software.

For Co-IP, the cells were lysed in IP cell lysis buffer (R0010; Solarbio) with phosphatase inhibitors and protease inhibitors. The supernatants, as cell lysates, were incubated with the appropriate antibody at 4 °C overnight. After the 4 h incubation with 50 μL protein A/G (sc-2003; Santa Cruz) at 4 °C, the precipitates were washed 4 times with wash buffer. The protein-bound beads were eluted by boiling in denaturing SDS-gel loading buffer and subsequently analyzed by WB.

### Reverse transcription quantitative polymerase chain reaction (RT-qPCR)

Total RNA was isolated by RNAiso Plus (9109; Takara). Based on the manufacturer’s instructions, complementary deoxyribonucleic acid (cDNA) was synthesized after genomic DNA erasure by a PrimeScript™ RT Reagent Kit (RR047A; Takara). RT-qPCR was conducted using the TB Green PCR Kit (RR820A; Takara) by Roche LightCycler 480II (Roche; Basel). The comparative CT method was used to measure the mRNA expression. The genes we detected were normalized to the GAPDH mRNA level. The primer sequences used for quantitative RT‒PCR are listed in Supplement Table 2.

### Plasmids and lentiviruses transfection

All plasmids were purchased from Shandong Gene&Bio Co. Ltd (Shandong, China). The human IRGM, either full-length or truncated variants, was subcloned into the pcDNA3.1 vector with a FLAG tag. The human KEAP1 cDNA was cloned with an HA tag. HEK293T cells were transiently transfected for 48 h with FLAG-vector, FLAG-IRGM, FLAG-IRGM-D1 (position 1–31), FLAG-IRGM-D2 (position 32–181), HA-vector, and HA-KEAP1, respectively. Then the cells were harvested and subjected to IP with anti-FLAG (20543-1-AP; Proteintech) or anti-HA (3724; Cell Signaling Technology) primary antibodies.

All lentiviruses for the experiment were purchased from Shanghai GeneChem Company. After RAW264.7 cells were infected with lentiviruses according to the manufacturer’s instructions, 4.5 μg/ml puromycin (P8230; Solarbio) was added to select the infected cells for 48 h. Then, DMEM with 10% FBS and 2 μg/ml puromycin was replaced to maintain stable gene-knocking or overexpression cell lines.

### Immunofluorescence staining

Cells were seeded on cover slips for 3 days with different treatments and permeabilized using 0.5% Triton X-100 after being fixed with 4% PFA. Cells were then blocked in 1% BSA at room temperature and cultured with the primary antibody at 4 °C overnight. After incubation with the specific secondary antibody (A23220; Abbkine) for 1 h, DAPI was stained for 5 min and visualized by fluorescence microscope.

### Intracellular ROS measurement

As in our previous study, ROS was examined by DCFH-DA probe (s0033; Beyotime) [25]. Cells were cultured with different treatments for 24 h and stimulated with 10 μM DCFH-DA in α-MEM at 37 °C for 20 min. After washing with α-MEM three times, fluorescence intensity was examined using a fluorescence microscope.

### Flow cytometry

RAW264.7 cells in different groups were incubated in 40 ng/ml RANKL for 24 h. Then, cells were harvested and resuspended with 10 μM DCFH-DA in α-MEM for 20 min at 37 °C. Cells were shaken every 5 min. Next, cells were washed three times with cold PBS and resuspended in 500 μL PBS. Fluorescence intensity was measured using flow cytometry with an excitation wavelength of 488 nm and an emission wavelength of 525 nm.

### Data source

The dataset of GSE176265 described the gene expression profiling during osteoclast differentiation treated with 100 ng/ml RANKL and 30 ng/ml M-CSF for 0, 1, 2, and 3 days. The data were collected from the GEO database (https://www.ncbi.nlm.nih.gov/geo/query/acc.cgi?acc=GSE176265) deposited by You JS et al. The original data from GSE176265 were analyzed to clarify the upregulated or downregulated genes of BMDMs on day 1, day 2, and day 3 mediated by RANKL, respectively [26]. The results were visualized in volcano diagrams. Then, we obtained the overlapping targets of upregulated genes on different days. A Venn diagram was acquired to identify the intersection of upregulated genes in different days via the Venn diagram website (https://bioinformatics.psb.ugent.be/webtools/Venn/) [27]. Using the Functional Annotation tool of Database for Annotation, Visualization and Integrated Discovery (DAVID) 6.8 (https://david.ncifcrf.gov/) [28], Kyoto encyclopedia of genes and genomes (KEGG) pathway analysis and Gene ontology (GO) enrichment analysis were performed with a corrected *p* < 0.05. Then the top ten GO items or KEGG pathways were selected and visualized by Graphpad software.

### Protein docking and molecular docking

The protein structure of Keap1 (Q9Z2X8) and Irgm1 (Q60766) was obtained from the AlphaFold Protein Structure Database (https://www.alphafold.ebi.ac.uk/) and using the GRAMM Web Server (https://gramm.compbio.ku.edu/gramm) for protein docking [29]. Next, Keap1 was considered as the receptor while Irgm1 was defined as the ligand. We obtained the docking results with the default parameters, and the lowest-energy docking pose was visualized using PyMol2.3.0 software. Docking energy was calculated using Hdock Server (http://hdock.phys.hust.edu.cn/) [30].

The 3D structure of mifamurtide was obtained from the PubChem database (https://pubchem.ncbi.nlm.nih.gov/) [31]. After removing water molecules and original ligands by Pymol 2.3.0, the target proteins were uploaded into Autodock Tools 1.5.6 for adding hydrogen, calculating charge, assigning charge, and specifying atom type, while the ligand was hydrogenated, charges calculated, charges assigned, and rotatable bonds set. Next, we defined the Grid Box size and the genetic algorithm, while simulating molecular docking and visualizing the docking results.

### Network proximity measure

By using the STRING database (https://cn.string-db.org/cgi/input.pl) [32], with IRGM as the keyword and the species as homo sapiens, the PPI network of IRGM-related genes was obtained. Subsequently, the IRGM-related genes from the STRING database were uploaded to Shengxin bean sprouts (http://www.sxdyc.com/drugGeneset) to predict potential drugs according to Niu et al.'s research [33]. In brief, the candidate drugs with a significantly shorter distance to IRGM-related genes and FDR < 0.001 were identified as potential therapeutic agents. Finally, the distance distribution map between IRGM-related proteins and drug targets was obtained.

### Statistical analysis

Experiments are conducted at least three times, and the data are expressed as the mean ± standard deviation (SD). For statistical analysis, means were compared using one-way ANOVA for comparisons between multiple groups, while the *T*-test was used for two-group comparisons. *p* < 0.05 is considered statistically significant. All data analyses were performed using Prism 9, and all quantitative values are expressed as the means ± SD.

## Results

### Irgm1 is correlated with osteoporosis and osteoclast differentiation

**Fig. 1:**
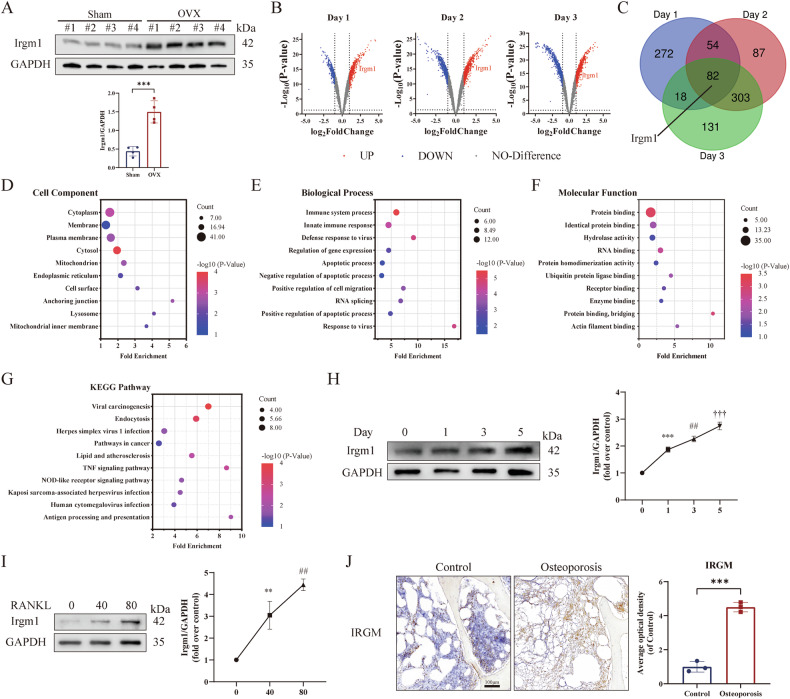
Irgm1 is correlated with osteoporosis and osteoclast differentiation. **A** WB showed the expression of Irgm1 in BMDMs. Quantitative data were normalized to GAPDH and presented as means ± SD. ^∗∗∗^*p* < 0.001 vs the indicated groups. **B** Volcano diagram displayed that Irgm1 was continuously upregulated during osteoclast differentiation mediated by RANKL at days 1, 2, and 3. **C** Venn diagram summarizes 82 candidate targets of RANKL-induced osteoclast differentiation at days 1, 2, and 3. **D**–**F** GO enrichment analysis to identify the top 10 significant terms of cell component (**D**), biological process (**E**), and molecular function (**F**), corresponding to the candidate targets. **G** KEGG pathway analysis suggested potential signaling pathways by which Irgm1 regulated osteoclast differentiation. **H** WB analysis of Irgm1 expression in RANKL-induced BMDMs. Cells were treated with RANKL (40 ng/ml) and M-CSF (10 ng/ml) for 0, 1, 3, and 5 days. Quantitative data were normalized to GAPDH and presented as means ± SD (*n* = 3). ^∗∗∗^*p* < 0.001, ^##^*p* < 0.01, and ^†††^*p* < 0.001 vs 0 group. **I** WB analysis of Irgm1 expression after treatment with different concentrations of RANKL (0, 40, 80 ng/ml) for 3 days. Quantitative data were normalized to GAPDH and presented as means ± SD (*n* = 3). ^∗∗^*p* < 0.01 and ^##^*p* < 0.01 vs 0 group. **J** Immunohistochemical staining images of bone tissues from the osteoporosis and healthy (Control) group. Scale bar = 100 μm. Quantitative analysis of the immunohistochemical staining and presented as means ± SD. ^∗∗∗^*p* < 0.001 vs the control group.

We first explored Irgm1 expression levels during osteoporosis induced by OVX. WB showed that Irgm1 protein levels in BMDMs were significantly upregulated in OVX mice (Fig. 1A). This result was further clarified by analyzing the dataset uploaded by You JS et al. (GSE176265). The database included different days (0, 1, 2, 3) of BMDMs treated with 100 ng/ml RANKL and 30 ng/ml M-CSF. As shown in Fig. 1B and C, Irgm1 was one of 82 key genes whose expression was continuously upregulated in osteoclast differentiation mediated by RANKL at days 1, 2, and 3 (Supplement Table 3).

To further detect the biological characteristics of these 82 key genes on osteoclastogenesis, GO enrichment and KEGG analysis were presented in Fig. 1D–G. After analyzing the terms according to the significance from *p* value and gene count, the results indicated that Irgm1 may regulate ubiquitin protein ligase protein binding and RNA binding in cytosol, cytoplasm, and plasma membrane to affect the defense response of the immune system of BMDMs, thereby promoting RANKL-mediated osteoclast differentiation (Fig. 1D–F). In addition, the KEGG pathway enrichment suggested that Irgm1 was correlated with the TNF signaling pathway and osteoclast differentiation (Fig. 1G).

To validate the role of Irgm1 on osteoclast differentiation, WB showed the protein level of Irgm1 in BMDMs stimulated with 40 ng/ml RANKL and 10 ng/ml M-CSF for different days of osteoclasts differentiation (0, 1, 3, 5 days), or treated with 10 ng/ml M-CSF and different concentration of RANKL (0, 40, 80 ng/ml) for 3 days. We found that Irgm1 expression exhibited a time-dependent pattern during osteoclast differentiation (Fig. 1H), and RANKL upregulated Irgm1 levels in a dose-dependent pattern (Fig. 1I). Additionally, we found that IRGM expression in the bone tissue was dramatically increased under osteoporosis conditions (Fig. 1J). Collectively, these data indicated that Irgm1 regulated the process of osteoclastogenesis, and its expression is positively correlated with osteoporosis.

### Targeted deletion of Irgm1 decreases OVX-induced bone loss

**Fig. 2:**
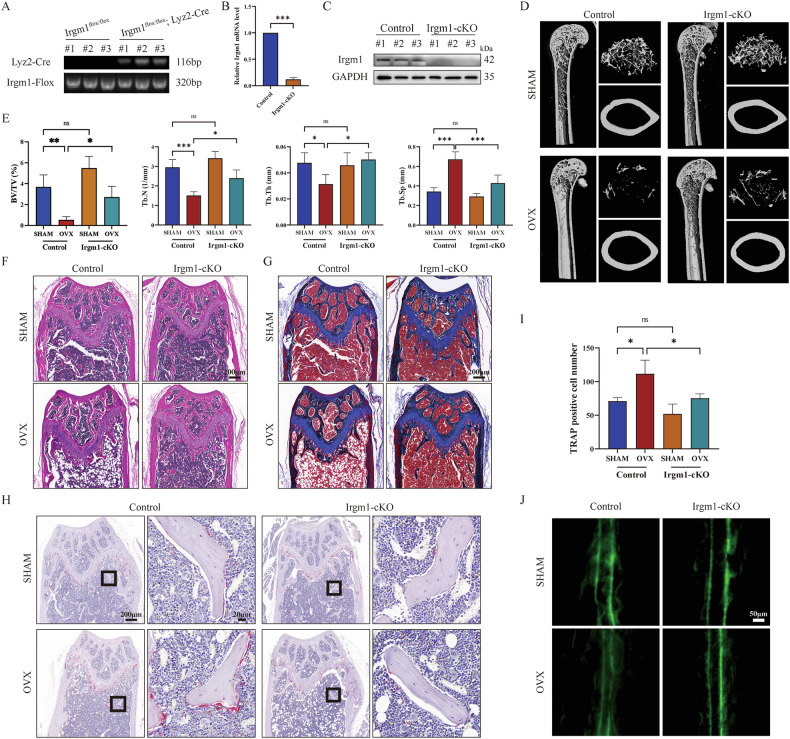
Targeted deletion of Irgm1 decreases OVX-induced bone loss. **A** Genotype analysis for Irgm1. **B** RT-qPCR demonstrated the mRNA levels of Irgm1 in BMDMs from Control and Irgm1-cKO mice. Quantitative data were normalized to GAPDH and presented as means ± SD (*n* = 3), ^∗∗∗^*p* < 0.001 vs the indicated groups. **C** WB analysis of Irgm1 level in BMDMs derived from Irgm1^flox/flox^ (Control) and Irgm1^flox/flox^; Lyz2-Cre (Irgm1-cKO) mice. **D** Representative three-dimensional (3D) reconstructed CT images of the distal femur from 5-month-old mice. **E** Quantitative measurements of BV/TV, Tb. N, Tb. Th, and Tb.Sp. All data were presented as the mean ± SD (*n* = 4), ^∗^*p* < 0.05, ^∗∗∗^*p* < 0.001, and ns (not statistically significant) vs the indicated groups. **F** Representative images of haematoxylin and eosin (H&E) staining on paraffin-embedded femur sections of the distal femur from 5-month-old mice from different groups. Scale bar = 200 μm. **G** Representative images of Masson staining on paraffin-embedded femur sections of the distal femur from 5-month-old mice from different groups. Scale bar = 200 μm. **H** Representative images showed TRAP activity staining in femur sections in different groups. Scale bar in lower magnification = 200 μm, scale bar in higher magnification = 20 μm. **I** Quantitative analysis of the number of TRAP-positive cells in femoral sections of different groups (*n* = 3), ^∗^*p* < 0.05 and ns vs the indicated groups. **J** Calcein double labeling of mineral layers of the femur.

To detect the potential role of Irgm1 in bone loss caused by OVX in vivo, we first distinguished Irgm1^flox/flox^ (control) and Irgm1^flox/flox^; Lyz2-Cre (Irgm1-cKO) mice through genotype analysis (Fig. 2A). Then, we confirmed that the expression of Irgm1 in BMDMs was apparently decreased in both mRNA (Fig. 2B) and protein (Fig. 2C) levels, suggesting that we successfully generated the Irgm1 deletion mouse model. The mice from different groups were sacrificed at 5-month-old. Micro-CT images confirmed the extensive bone loss in OVX mice from the control group compared with sham mice. Notably, the OVX mice from the Irgm1-cKO group showed an apparently increased bone mass compared with OVX mice from the control group, indicating that the depletion of Irgm1 rescued the OVX-induced osteoporosis (Fig. 2D). Quantitative micro-CT measurements illustrated an increase in BV/TV, Tb.N, and Tb.Th, whereas Tb.Sp showed the opposite in OVX mice from the Irgm1-cKO group (Fig. 2E). Moreover, compared with the control group, sham mice of the Irgm1-cKO group showed a slight increase in bone mass, but they were not statistically different. H&E stain (Fig. 2F) and Masson stain (Fig. 2G) sections both demonstrated that the trabecular bone mass of Irgm1-cKO mice was significantly increased under the OVX-induced osteoporosis compared with the control group.

To confirm whether the high bone mass was the result of a reduced number of osteoclasts or an increased number of osteoblasts in vivo, we determined the cell numbers for TRAP staining. As shown in Fig. 2H, I, the number of TRAP-positive multinucleated cells was notably decreased in Irgm1-cKO mice compared with the control group. In addition, the double calcein labeling assay demonstrated that the Irgm1 deficiency showed more calcium deposition, indicating Irgm1-cKO stimulated bone formation and enhanced osteoporotic osteogenesis (Fig. 2J). These results supported the role of Irgm1 in both osteoclasts and osteoblasts, as well as highlighted the importance of Irgm1 in bone remodeling.

### Irgm1 deficiency attenuates osteoclast differentiation and function

**Fig. 3:**
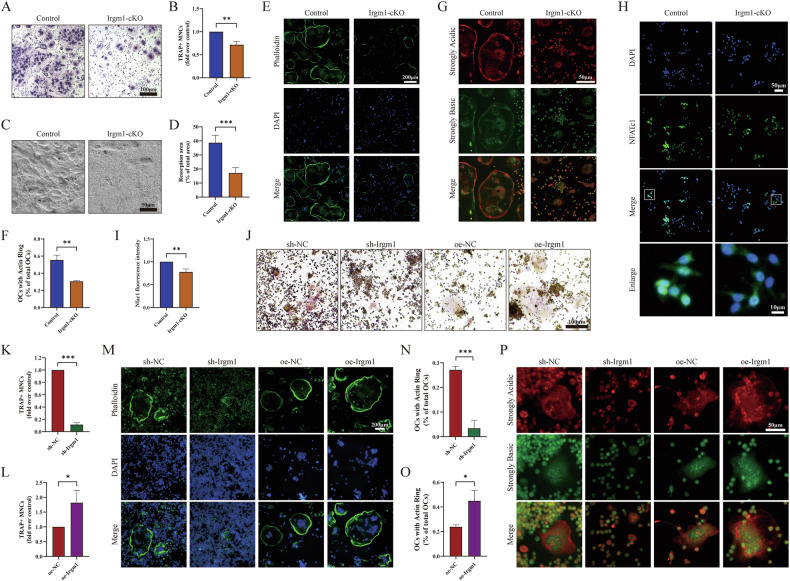
Irgm1 deficiency attenuates osteoclast formation and bone resorptive activity. **A** TRAP staining of osteoclasts differentiated from BMDMs obtained from Control and Irgm1-cKO mice. BMDMs were subjected to osteoclast full differentiation induced with RANKL (40 ng/ml) and M-CSF (10 ng/ml) for 5 days. Scale bar = 100 μm. **B** Quantitative analysis of TRAP-positive cells (contains three or more nuclei) (MNCs) from (**A**), and data were presented as the means ± SD (*n* = 3). ^∗∗^*p* < 0.01 vs the indicated group. **C** Bone resorption activity of osteoclasts derived from Control and Irgm1-cKO BMDMs cultured on bovine cortical bone slice. Scale bar = 50 μm. **D** Quantitative analysis of results from (**C**) (*n* = 3). ^∗∗∗^*p* < 0.001 vs the indicated groups. **E** F-actin ring stain. BMDMs were stained with phalloidin and DAPI and visualized by fluorescence microscope. Scale bar = 200 μm. **F** The number of quantitative data from (**E**) (*n* = 3). ^∗∗^*p* < 0.01 vs the indicated groups. **G** Acidified compartments in osteoclasts were stained with acridine orange (AO) dye and visualized by fluorescence microscope. Scale bar = 50 μm. **H** Immunofluorescence analysis of Nfatc1 expression in BMDMs subjected to osteoclast induction for 3 days. Scale bar in lower magnification = 50 μm, scale bar in higher magnification = 10 μm. **I** Quantitative analysis of results from (**H**) (*n* = 3). ^∗∗^*p* < 0.01 vs the indicated groups. **J** TRAP staining of RAW264.7. Cells were transfected with Irgm1 knockdown or overexpression lentivirus and induced with 40 ng/ml RANKL for 5 days. Scale bar = 100 μm. **K**, **L** Quantitative analysis of TRAP-positive MNCs from (**J**) (*n* = 3). ^∗^*p* < 0.05 and ^∗∗∗^*p* < 0.001 vs the indicated groups. **M** F-actin ring stain. RAW264.7 were stained with phalloidin and DAPI and visualized by fluorescence microscope. Scale bar = 200 μm. **N**, **O** The number of quantitative results from (**M**) (*n* = 3). ^∗^*p* < 0.05 and ^∗∗∗^*p* < 0.001 vs the indicated group. **P** AO staining was visualized by a fluorescence microscope. Scale bar = 50 μm.

To demonstrate the effect of Irgm1 in osteoclastogenesis, BMDMs isolated from control and Irgm1-cKO mice were differentiated using 40 ng/ml RANKL and 10 ng/ml M-CSF for 5 days. Compared with the control group, Irgm1-deficient precursor cells showed a weaker osteoclast differentiation potential, with fewer TRAP-positive multinucleated osteoclasts and significantly smaller (Fig. 3A, B). We then evaluated the bone resorption function of osteoclasts and verified that Irgm1-deficient osteoclasts had a significantly reduced ability to resorb bovine cortical bone slices (Fig. 3C, D). F-actin ring formation was also observed because this is essential for osteoclast function [34]. Irgm1-deficient osteoclasts displayed a smaller ring structure (Fig. 3E, F). Acridine orange stain is used to assess osteoclastic acidification. Less red fluorescence was shown in Irgm1-deficient mature osteoclasts, suggesting that Irgm1 deficiency inhibited the acidified compartments (Fig. 3G). Furthermore, the nuclear localization of Nfatc1 was tested by immunofluorescence. Loss of Irgm1 weakened the fluorescence intensity of Nfatc1 and decreased the level in the nucleus (Fig. 3H, I).

Based on the Irgm1 knockout model, we generated an Irgm1 knockdown and overexpression osteoclast precursor model, which were stably transfected in RAW264.7 cells, respectively. Irgm1 knockdown (sh-Irgm1) (Supplement Fig. 1A–C) and overexpression (oe-Irgm1) (Supplement Fig. 1D–F) in RAW264.7 were respectively demonstrated by WB and RT-qPCR. Via CCK-8 assay, there was no effect on the viability of knocking down Irgm1 in RAW264.7 cells compared with group sh-NC at 3 and 5 days no matter cultured with DMEM (Supplement Fig. 1G) or a-MEM (Supplement Fig. 1H) medium. We next evaluated whether Irgm1 could regulate osteoclast differentiation and bone resorptive function by using these two cell models. Knocking down Irgm1 significantly decreased the generation of osteoclasts. In contrast, overexpression of Irgm1 significantly increased the number of osteoclasts compared with the oe-NC group (Fig. 3J–L). In addition, both of F-actin rings and size were reduced in the depletion of the Irgm1 group, while overexpression of Irgm1 significantly promoted the formation of rings (Fig. 3M–O). It is also found by AO stain that the Irgm1 regulates the secretion of acidic vesicles in osteoclasts (Fig. 3P). Therefore, our findings demonstrated the importance of Irgm1 in regulating osteoclast differentiation and function, consistent with the osteoporotic phenotype detected in the Irgm1-deficient mouse model.

### Irgm1 mediates the changes in osteoclast marker genes by regulating ROS

**Fig. 4:**
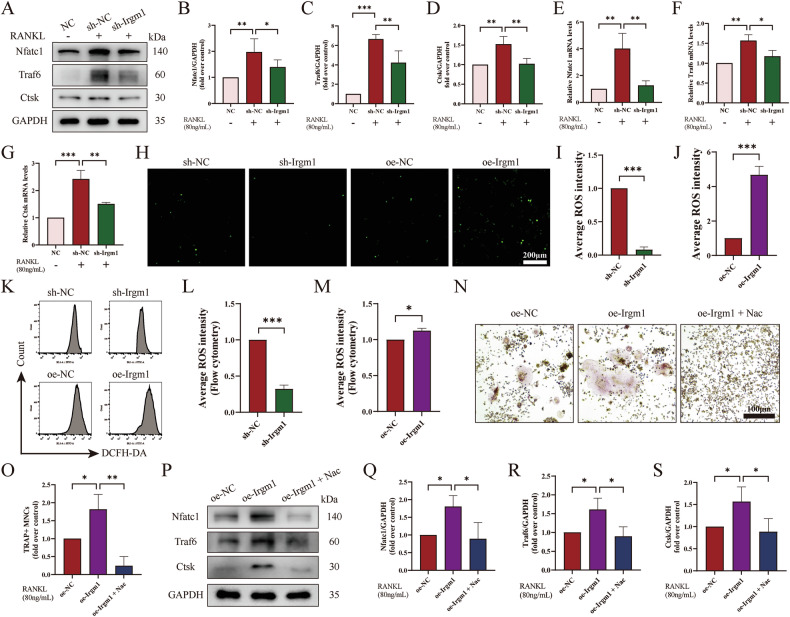
Irgm1 mediates the changes in osteoclast marker genes by regulating ROS. **A** WB analysis of Nfatc1, Traf6, and Ctsk in protein levels in osteoclasts derived from RAW264.7 cells after different stimulation for 3 days. **B**–**D** Quantitative analysis of Nfatc1 (**B**), Traf6 (**C**), and Ctsk (**D**) from (**A**) was normalized to GAPDH and presented as means ± SD (*n* = 3). ^∗^*p* < 0.05, ^∗∗^*p* < 0.01, and ^∗∗∗^*p* < 0.001 vs the indicated groups. **E**–**G** RT-qPCR demonstrated the mRNA levels of Nfatc1 (**E**), Traf6 (**F**), and Ctsk (**G**) in osteoclasts with different treatments. Quantitative data were normalized to GAPDH and presented as means ± SD (*n* = 3), ^∗^*p* < 0.05, ^∗∗^*p* < 0.01, and ^∗∗∗^*p* < 0.001 vs the indicated groups. **H** DCFH-DA probe labeled intracellular ROS in RAW264.7. Cells from different groups treated with 40 ng/ml RANKL for 24 h were detected by DCFH-DA probe and visualized by fluorescence microscope. **I**, **J** Quantitative results from (**H**) (*n* = 3). ^∗∗∗^*p* < 0.001 vs the indicated group. **K** Flow cytometry measured the density of DCFH-DA in RAW264.7 cells from different groups after 24 h of treatment. **L**, **M** Quantitative results from (**K**) (*n* = 3). ^∗^*p* < 0.05 and ^∗∗∗^*p* < 0.001 vs the indicated groups. **N** TRAP staining of osteoclast differentiated from RAW264.7. Cells from different groups were treated with or without 5 mM Nac for 5 days under 40 ng/ml RANKL conditions. Scale bar = 100 μm. (**O**) Quantitative analysis of TRAP-positive MNCs from (**N**) (*n* = 3). ^∗^*p* < 0.05 and ^∗∗^*p* < 0.01 vs the indicated group. **P** WB analysis of expression of Nfatc1, Traf6, and Ctsk in RAW264.7 from different groups of cells treated with or without 5 mM Nac for 3 days under 40 ng/ml RANKL conditions. **Q**–**S** Quantitative analysis of Nfatc1 (**Q**), Traf6 (**R**), and Ctsk (**S**) from (**P**) was normalized to GAPDH and presented as means ± SD (*n* = 3). ^∗^*p* < 0.05 vs the indicated groups.

To investigate the mechanism behind the inhibition of RANKL-mediated osteoclast formation and function that resulted from Irgm1 deficiency, we tested the osteoclast marker genes, including Nfatc1, Traf6, and Ctsk, at both mRNA and protein levels. WB confirmed that the levels of osteoclast genes were effectively suppressed, caused by the deficiency of Irgm1 (Fig. 4A–D). These results were further verified at mRNA levels by RT-qPCR (Fig. 4E–G).

Overproduction of ROS causes oxidative stress, wherein the elevated formation of ROS is the key factor of osteoclast activation [35]. To assess the role of Irgm1 on ROS formation induced by RANKL, the fluorescent probe DCFH-DA was used. We found that the generation of intracellular ROS was significantly reduced when Irgm1 was knocked down compared with the sh-NC group, whereas overexpression of Irgm1 robustly increased the fluorescence intensity of DCFH-DA (Fig. 4H–J). These results were further confirmed using flow cytometry analysis (Fig. 4K–M). To better understand the effects of Irgm1 on ROS-induced osteoclastogenesis, ROS scavenger Nac was stimulated in Irgm1-overexpressing RAW264.7 cells. As expected, Irgm1-induced excessive activation of osteoclasts was reversed by Nac treatment (Fig. 4N, O). These results were further verified by WB at the protein levels (Fig. 4P–S), suggesting Irgm1 inhibited osteoclastogenesis mainly by suppressing intracellular ROS production.

### Irgm1 regulates the Keap1-Nrf2 axis to amplify RANKL-induced oxidative assaults

**Fig. 5:**
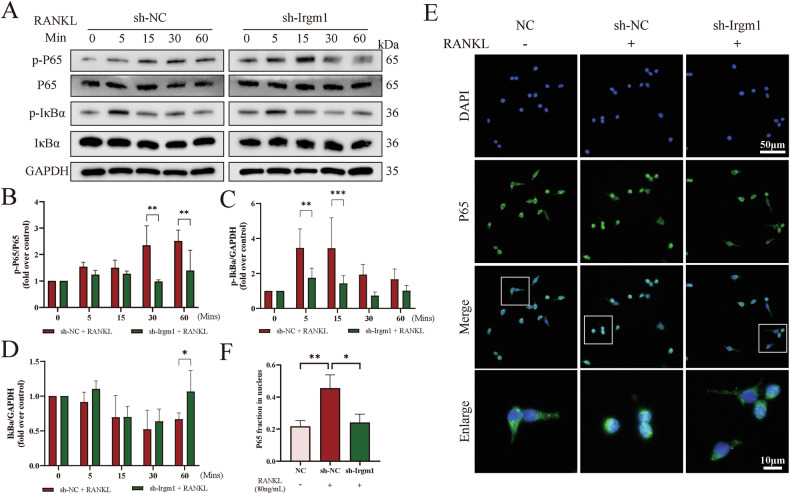
Irgm1-deficient inhibits the RANKL-induced NF-κB signaling. **A** WB detected phosphorylated or total P65 and IκB in transfected RAW264.7 cells induced with 40 ng/ml RANKL for the indicated times. **B**–**D** Quantitative analysis of phosphorylated P65 (**B**), phosphorylated IκB (**C**), and total IκB (**D**) from (**A**) was normalized to GAPDH and presented as means ± SD (*n* = 3). ^∗∗^*p* < 0.01, and ^∗∗∗^*p* < 0.001 vs the indicated groups. **E** Immunofluorescence analysis of P65 level in different groups of RAW264.7 for 3 days. Scale bar in lower magnification = 50 μm, scale bar in higher magnification = 10 μm. **F** Quantitative results from (**E**) (*n* = 3). ^∗^*p* < 0.05 and ^∗∗^*p* < 0.01 vs the indicated groups.

To explore the deeper mechanisms behind the protection conferred by Irgm1 deletion, we first verified NF-κB activation, which plays a predominant role in osteoclastogenesis. We found that the loss of Irgm1 significantly inhibited phosphorylation of P65 after 30 min RANKL stimulation, while phosphorylation of IκBα was reduced at 5 min and 15 min after RANKL treatment compared with the sh-NC group. However, the effect on the degradation of IκBα did not occur until 60 min (Fig. 5A–D). Immunofluorescence staining showed that Irgm1-deficient cells reversed the increase of RANKL-induced nucleus translocation of P65 (Fig. 5E, F). These results detected that the NF-κB signaling pathway (the downstream of TNF and NOD-like receptor signaling pathway) was suppressed by Irgm1-deficient, supporting the postulated from network pharmacology analysis (Fig. 1G).

**Fig. 6:**
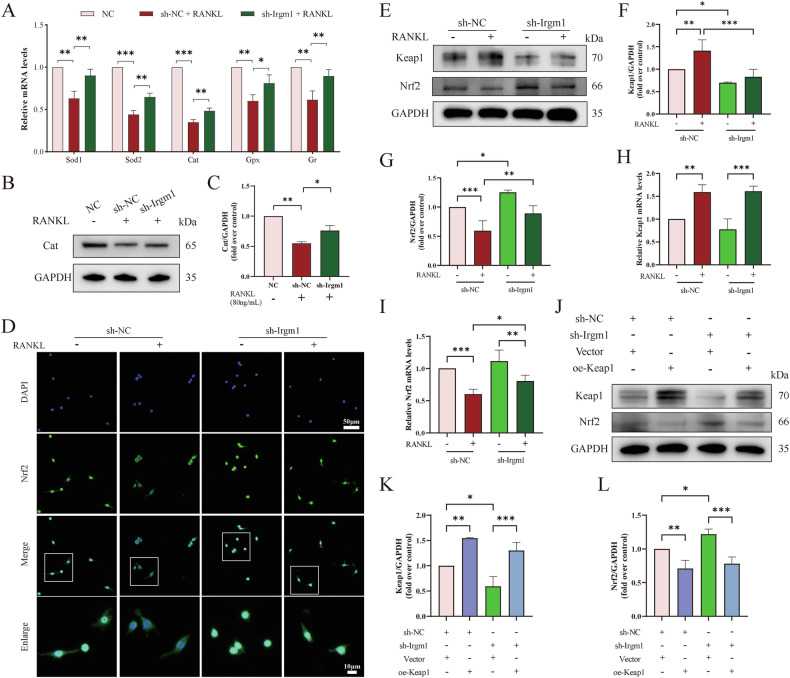
Irgm1 regulates the Keap1-Nrf2 axis to amplify RANKL-induced oxidative assaults. **A** RT-qPCR detected the mRNA levels of Sod1, Sod2, Cat, Gpx, and Gr in RAW264.7 under different treatments. Quantitative data were normalized to GAPDH and presented as means ± SD (*n* = 3), ^∗^*p* < 0.05, ^∗∗^*p* < 0.01, and ^∗∗∗^*p* < 0.001 vs the indicated groups. **B** WB showed the expression of Cat in RAW264.7 under different treatments for 3 days. **C** Quantitative analysis of Cat was normalized to GAPDH and presented as means ± SD (*n* = 3). ^∗^*p* < 0.05 and ^∗∗^*p* < 0.01 vs the indicated groups. **D** Immunofluorescence analysis of Nrf2 level in RAW264.7 under different stimuli for 3 days. Scale bar in lower magnification = 50 μm, scale bar in higher magnification = 10 μm. **E** WB showed the expression of Keap1 and Nrf2 in RAW264.7 under different treatment for 3 days. **F**, **G** Quantitative analysis of Keap1 (**F**) and Nrf2 (**G**) from (**E**) were normalized to GAPDH and presented as means ± SD (*n* = 3). ^∗^*p* < 0.05, ^∗∗^*p* < 0.01, and ^∗∗∗^*p* < 0.001 vs the indicated groups. **H**, **I** mRNA levels of Keap1 (**H**) and Nrf2 (**I**) under different conditions. Quantitative data were normalized to GAPDH and presented as means ± SD (*n* = 3), ^∗^*p* < 0.05, ^∗∗^*p* < 0.01, and ^∗∗∗^*p* < 0.001 vs the indicated groups. **J** WB showed Keap1 and Nrf2 expression in RAW264.7 transfected with different reagents. **K**, **L** Quantitative analysis of Keap1 (**K**) and Nrf2 (**L**) from (**J**) were normalized to GAPDH and presented as means ± SD (*n* = 3). ^∗^*p* < 0.05, ^∗∗^*p* < 0.01, and ^∗∗∗^*p* < 0.001 vs the indicated groups.

We continued to verify the reply of antioxidant expression to confirm whether the ROS defense system was mediated by Irgm1. Using the RT-qPCR assay, we found that Irgm1-deficient reversed the low mRNA levels of antioxidant genes (Sod1, Sod2, Cat, Gpx, and Gr) induced by RANKL (Fig. 6A). Consistently, Cat showed the same results at the protein level by WB assay (Fig. 6B, C). Next, we evaluated the level of Nrf2, a key protein in resisting oxidative stress induced by RANKL [36]. The immunofluorescence results demonstrated that the Nrf2 level reduced in RANKL-stimulated cells, wherein the nucleus translocation of Nrf2 was significantly increased in Irgm1-deficient cells (Fig. 6D). The protein expression levels of Nrf2 and Keap1 via WB were examined (Fig. 6E–G). We found that the absence of Irgm1 apparently increased the Nrf2 level even before RANKL treatment and increased after the RANKL stimulation. Not surprisingly, Keap1 showed the opposite trend in protein levels. Via RT-qPCR, similar results were revealed at the mRNA level of Nrf2 and Keap1 (Fig. 6H, I). Interestingly, the mRNA level of Keap1 did not display any difference between the sh-NC and sh-Irgm1 group with or without RANKL treatment (Fig. 6H), indicating that Irgm1 may promote Keap1 by a post-translational mechanism. Furthermore, Keap1 expression level is considered a key factor in determining the ubiquitination of Nrf2 [12]. Based on these reports, we demonstrated that overexpression of Keap1 could restore the effects of Irgm1 deletion on Nrf2 expression, suggesting that Irgm1 negatively regulated Nrf2 through Keap1 (Fig. 6J–L). These data demonstrated that Irgm1 negatively regulated Nrf2 by impacting Keap1.

### Irgm1 protects Keap1 from ubiquitination and degradation

**Fig. 7:**
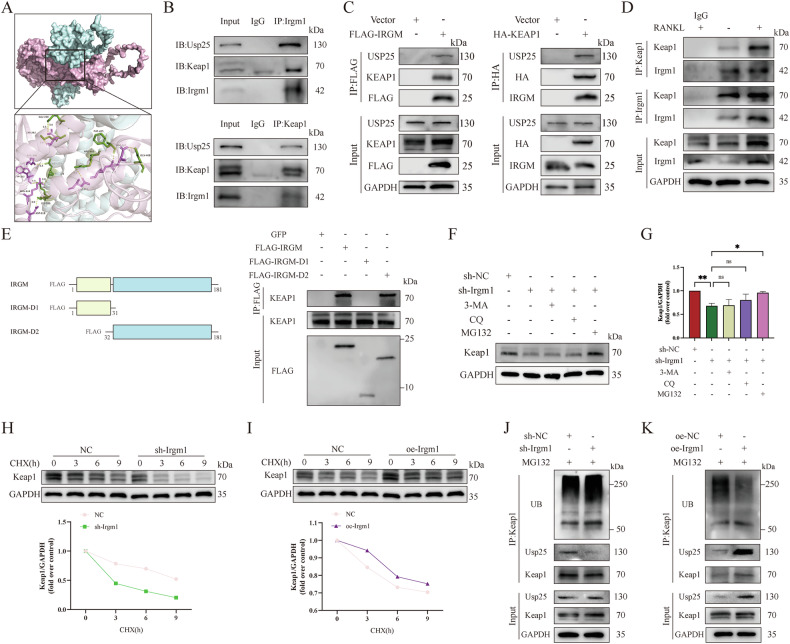
Irgm1 protects Keap1 from ubiquitination and degradation. **A** Up: protein-protein docking simulation of Irgm1 (palecyan) and Keap1 (lightpink). Down: the key amino acids from Irgm1 (green) and Keap1 (pink) in binding. Yellow dashed lines indicate the hydrogen bonds. **B** Immunoprecipitation showed the interaction between endogenous Irgm1, Keap1, and Usp25 in RAW264.7 cells. **C** Co-IP analysis of the interaction between endogenous Irgm1, Keap1, and Usp25 in HEK293T cells transfected with FLAG-IRGM and HA-KEAP1. **D** Co-IP analysis of the interaction between Irgm1 and Keap1 in BMDMs induced with or without 40 ng/ml RANKL for 24 h. **E** Mapping the domains in IRGM that interact with KEAP1. FLAG-tagged domain 1 (D1) and D2 of IRGM were expressed in HEK293T cells and immunoprecipitated. The immunoprecipitates were then analyzed for the presence of KEAP1. **F** WB showed Keap1 expression in RAW264.7. Cells were pretreated with 5 μM MG132 or 20 μM CQ or 1 mM 3-MA for 2 h and then stimulated with 40 ng/ml RANKL for 24 h. **G** Quantitative results from **F** were normalized to GAPDH and presented as means ± SD (*n* = 3). ^∗^*p* < 0.05, ^∗∗^*p* < 0.01, and ns (not statistically significant) vs the indicated groups. **H**, **I** WB showed Keap1 levels in RAW264.7 under different situations. Cells transfected with Irgm1 knockdown lentivirus (**H**), or overexpression lentivirus (**I**) were treated with 50 ng/ml cycloheximide (CHX) for the indicated times to measure the half-lives of keap1. Quantitative data were normalized to GAPDH and presented as means ± SD (*n* = 3). **J**, **K** WB showed the ubiquitination expression of endogenous Keap1 and the expression of Usp25 in RAW264.7 cells depleted of Irgm1 (**J**) or in RAW264.7 cells overexpressing Irgm1 (**K**). Cells were treated with 5 μM MG132 and 40 ng/ml RANKL for 6 h.

To identify Irgm1 interactors, we first used protein-protein docking to predict Irgm1's probable interaction with Keap1, which revealed the formation of nine hydrogen bonds between Irgm1 and Keap1, indicating the high binding activity (Fig. 7A). Hdock results confirmed that the binding energy of Irgm1 and Keap1 was −256 kcal/mol. To further validate the interaction of Irgm1 and Keap1, Co-IP analysis was performed from extracts of RAW264.7 cells overexpressing Irgm1 or Keap1. We immunoprecipitated Irgm1 or Keap1 and checked the presence of the other in the precipitates. Much to our satisfaction, Irgm1 could bring down Keap1, and vice versa. Furthermore, Co-IP results confirmed the presence of Irgm1, Keap1, and Usp25 in the same protein complex in RAW264.7 (Fig. 7B). Similar results were further validated in HEK293T cells by Co-IP (Fig. 7C). To verify that the interaction might occur in BMDMs, we performed immunoprecipitation of endogenous Irgm1 or Keap1. As shown in Fig. 7D, the Co-IP further confirmed the interaction ability, and the interaction strength seemed to increase after RANKL stimulation, but that could just reflect the current upregulated expression levels of both Irgm1 and Keap1. To further describe the interaction between IRGM and KEAP1, we generated two truncations of IRGM and expressed these fragments in HEK293T cells to map the functional regions mediating their molecular interaction. The results showed that it is the IRGM-D2 domain that interacts with KEAP1, rather than the IRGM-D1 domain (Fig. 7E). These results verified that there was a direct or indirect physical interaction between Irgm1 and Keap1.

Next, we combined the results in Fig. 6H, the protein (but not mRNA) level of Keap1 was reduced in the loss of Irgm1 with or without RANKL treatment. Therefore, we sought to explore whether Irgm1 affects Keap1 stability associated with degradation. RAW264.7 cells were pretreated with 5 μM MG132 (proteasome inhibitor) or 20 μM CQ (lysosomal inhibitors), or 1 mM 3-MA (autophagy inhibitor) for 2 h and then stimulated with 40 ng/ml RANKL for 24 h. We determined that the decreased protein level of Keap1 induced by Irgm1-deficiency could only be rescued by MG132, indicating Keap1 is degraded by the proteasome (Fig. 7F, G). Moreover, we confirmed that the loss of Irgm1 shortened Keap1 half-life by using 50 ng/ml CHX (Fig. 7H), whereas Irgm1 overexpression extended the half-life (Fig. 7I). Consistent with this, we found that Keap1 was less associated with Usp25 in Irgm1-deficient RAW264.7 cells, resulting in a higher ubiquitination of Keap1 than in the sh-NC cells (Fig. 7J). Moreover, when Irgm1 was overexpressed, there was much less ubiquitination on Keap1, which may be due to the binding of Keap1 to more Usp25 (Fig. 7K). Taken together, these results suggested that Irgm1 protected Keap1 from ubiquitination and degradation.

### Pharmacological inhibition of Irgm1 alleviates OVX-induced osteoporosis

**Fig. 8:**
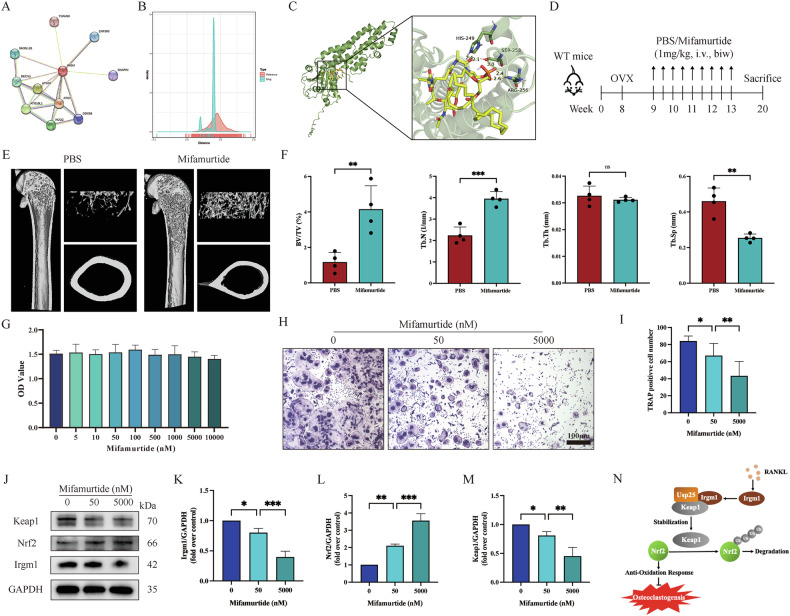
Pharmacological inhibition of Irgm1 alleviates OVX-induced osteoporosis. **A** The protein-protein (PPI) network of IRGM-related genes. **B** Network proximity analysis of the distribution of distances between drug targets and IRGM-related proteins. **C** Molecular docking diagram between mifamurtide and Irgm1. **D** Schematic of the experiment. Mice treated with mifamurtide (1 mg/kg, i.v.) or PBS one week after OVX were sacrificed at 20-week-old. **E** Representative 3D reconstructed CT images of the distal femur. **F** Quantitative measurements of BV/TV, Tb. N, Tb. Th, and Tb.Sp. All bar graphs are presented as the mean ± SD (*n* = 4), ^∗∗^*p* < 0.01, ^∗∗∗^*p* < 0.001, and ns vs the indicated groups. **G** CCK8 assay detected BMDMs viability with different concentrations of mifamurtide (*n* = 3). **H** TRAP staining of osteoclasts differentiated from BMDMs. BMDMs were subjected to osteoclast full differentiation induced with RANKL (40 ng/ml) and M-CSF (10 ng/ml) for 5 days under different concentrations of mifamurtide (0, 50, 5000 nM). Scale bar = 100 μm. **I** Quantitative analysis of TRAP-positive MNCs from (**H**) (*n* = 3). ^∗^*p* < 0.05 and ^∗∗^*p* < 0.01 vs the indicated group. **J** WB analysis of expression of Keap1, Nrf2, and Irgm1 in BMDMs induced with RANKL (40 ng/ml) and M-CSF (10 ng/ml) for 3 days, treated with different concentrations of mifamurtide (0, 50, 5000 nM). **K**–**M** Quantitative analysis of Keap1 (**J**), Nrf2 (**K**), and Irgm1 (**L**) from (**I**) were normalized to GAPDH and presented as means ± SD (*n* = 3). ^∗^*p* < 0.05, ^∗∗^*p* < 0.01 and ^∗∗∗^*p* < 0.001 vs the indicated groups. **N** Schematic illustration of the role of Irgm1 in regulating the Keap1-Nrf2 signaling pathway to oppose OVX-induced osteoporosis.

To obtain potential compounds targeting Irgm1, we first screened 11 IRGM-related genes from the STRING database (Fig. 8A and Supplement Table 4). By using network proximity, we obtained 17 candidate drugs related to the IRGM-related gene sets and mapped the distance distribution ranging from −2.25 to 1.95 (Fig. 8B and Supplement Table 5). The results showed that Mifamurtide (compound DB13615) was the candidate drug with the lowest distance value of −2.25, suggesting it may be an effective inhibitor of irgm1. To confirm how mifamurtide targets Irgm1 protein, the molecular docking was conducted. The results showed that mifamurtide formed close contact with various amino acid residues (hydrogen bonding with ARG-256, SER-253 and HIS-249) with a docking fraction of −5.3 kcal/mol, indicating the two showed good affinity (Fig. 8C). To further verify the in vivo effectiveness of mifamurtide, we treated mice with mifamurtide (1 mg/kg, i.v.) or PBS one week after OVX (Fig. 8D). Micro-CT images showed the bone mass in the mifamurtide group was significantly increased compared with PBS group (Fig. 8E). Quantitative micro-CT measurements illustrated increased in BV/TV and Tb.N, whereas Tb.Sp showed the opposite (Fig. 8F). Via CCK8 assay, there was no statistically significant difference in BMDMs with the treatment of different concentrations of mifamurtide, indicating that concentrations below 10,000 nM were safe (Fig. 8G). Furthermore, TRAP staining was conducted to verify the mechanism of mifamurtide in maintaining bone mass. The results showed that mature osteoclasts were significantly reduced under the simulation of mifamurtide, and the concentration of mifamurtide was inversely proportional to the number of mature osteoclasts (Fig. 8H, I). WB analyzed the effects of mifamurtide targeting Irgm1 on redox signaling during osteoclast differentiation. The results showed that mifamurtide inhibited the expression of irgm1 in a dose-dependent manner, which confirmed the results of drug screening. In addition, mifamurtide activated the Keap1-Nrf2 redox pathway in a dose-dependent manner (Fig. 8J–M). These data strongly supported that mifamurtide showed a potential role in alleviating OVX-induced osteoporosis by targeting Irgm1.

### Irgm1-deficient macrophage-derived medium promotes osteogenic differentiation

**Fig. 9:**
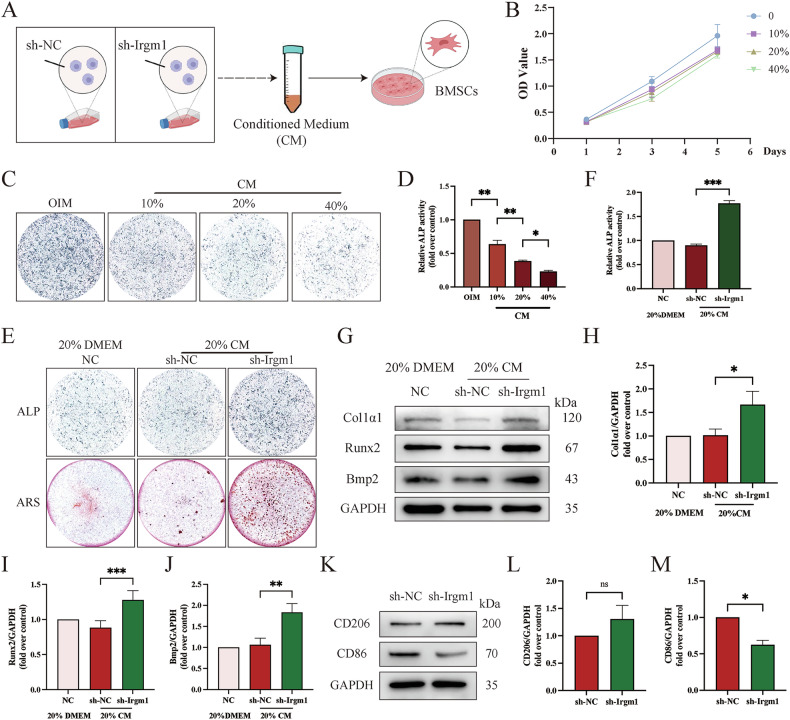
Irgm1-deficient macrophage-derived medium promotes osteogenic differentiation of BMSCs. **A** Schematic diagram showed RAW264.7 cells under different conditions. **B** CCK8 assay detected BMSCs viability with different percentages of conditioned medium of RAW264.7 cells for the indicated times (*n* = 3). **C**, **D** ALP staining (**C**) and ALP activity (**D**) of BMSCs under various treatments for 7 days. Cells were stimulated as previously described. **E**, **F** ALP and alizarin red stain (**E**) and ALP activity (**F**) of BMSCs under different conditions. BMSCs were cultured in OIM with 20% conditioned medium from transfected RAW264.7 cells for 7 days (**E**, upper panel) or 21 days (**E**, lower panel). **G** WB analysis of Col1a1, Runx2, and Bmp2 expression in BMSCs treated with OIM containing 20% conditioned medium from transfected RAW264.7 cells for 5 days. **H**–**J** Quantitative analysis of Col1a1 (**H**), Runx2 (**I**), and Bmp2 (**J**) from (**G**) were normalized to GAPDH and presented as means ± SD (*n* = 3). ^∗^*p* < 0.05, ^∗∗^*p* < 0.01, and ^∗∗∗^*p* < 0.001 vs the indicated groups. **K** WB analysis of the level of CD206 and CD86 in RAW264.7 cells. **L**, **M** Quantitative analysis of CD206 (**L**) and CD86 (**M**) from (**K**) were normalized to GAPDH and presented as means ± SD (*n* = 3). ^∗^*p* < 0.05 and ns vs the indicated groups.

Considering the positive role of Irgm1 in promoting macrophage differentiation into osteoclasts, and potential role in immune system process (Fig. 1E), we therefore explored whether Irgm1 is critical in bone immunity and promotes osteogenic differentiation. BMSCs were indirectly co-cultured with macrophages to detect the effect of macrophage-derived medium on bone formation of BMSCs (Fig. 9A). BMSCs were treated in their respective conditioned media. Via the CCK8 assay, the BMSCs' viability displayed a slight decrease with the treatment of conditioned medium (CM). But there was no statistical significance at each group in days 1–5, indicating the percentage of conditioned medium below 40% was safe to use on BMSCs (Fig. 9B). As shown in Fig. 9C, D, ALP staining verified that the osteogenic ability of BMSCs decreased with the increasing percentage of CM. Therefore, we selected the intermediate concentration (20%) as the baseline to respond to up- or down-regulation of cellular function in subsequent experiments. Then, we confirmed the osteogenesis of BMSCs treated with 20% conditioned medium from Irgm1-deficient or sh-NC RAW264.7 cells via ALP stain and Alizarin S stain. Surprisingly, ALP expression and mineralization nodules formation were apparently enhanced in the Irgm1-deficient group compared with the sh-NC group (Fig. 9E, F). Similar results were further verified at the protein level via WB (Fig. 9G–J), suggesting a positive impact of 20% conditioned medium from Irgm1-deficient RAW264.7 on promoting osteogenic differentiation.

It is well known that M2 macrophage polarization may contribute to osteoblast differentiation [37]. We then validated whether the promotion of the osteogenic ability of BMSCs was induced by the polarization of RAW264.7 cells. WB showed that M1 macrophages' surface antigen CD86 level significantly reduced, while the expression of M2 macrophages' surface antigen CD206 increased slightly inthe Irgm1 deficiency group (Fig. 9K–M). Taken together, Irgm1-depletion RAW264.7 cells' conditioned medium enhances the bone formation of BMSCs due to the number of M1 polarized macrophages.

## Discussion

The present study first demonstrated the important role of Irgm1 in the development of OVX-mediated osteoporosis by Micro-CT, H&E staining, Masson staining, and TRAP staining. In the absence of estrogen, macrophage-specific Irgm1 knockout mice showed a slower bone loss, which was caused by a reduced osteoclast number. Furthermore, osteoclast precursor cells isolated from these mice showed suppressed numbers and size of mature osteoclasts. Consistent with other studies, Irgm1 deficiency reduced F-actin ring formation, thereby affecting the adhesion and motility of osteoclast precursor cells [20]. Subsequently, osteoclast acidic vesicles failed to secrete into the sealing area of the F-actin ring, causing the acidified compartment to be unable to accumulate normally, resulting in weakened bone resorption function [11, 38]. In addition, in vitro experiments, RAW264.7 cells with Irgm1 loss using lentivirus showed reduced osteoclastogenesis and bone resorption activity mediated by RANKL. Moreover, Irgm1-deficient mice downregulated the levels of osteoclast-specific markers, including Nfatc1, Traf6, and Ctsk, at both mRNA and protein levels. In contrast, overexpression of Irgm1 in RAW264.7 apparently promoted osteoclast differentiation via increasing the number and size of mature osteoclasts, thereby facilitating bone resorption activity. These results demonstrated the potential therapeutic role of Irgm1 deficiency on OVX-mediated osteoporosis for the first time.

Based on our previous study, RANKL-mediated intracellular ROS accumulation plays an important role in the biological function of osteoclasts [11]. For all we know, increased ROS is the fundamental mechanism of the bone loss and strength [35, 39, 40]. In our current study, Irgm1 deficiency significantly reduced the generation of intracellular ROS, whereas overexpression of Irgm1 caused excessive accumulation of ROS in cells. However, Nac, as a scavenger of ROS, rescued osteoclast overactivation by scavenging excess ROS produced mediated by overexpression of Irgm1 [41]. Therefore, Irgm1 promoted RANKL-mediated osteoclastogenesis via regulating the accumulation of ROS in osteoclast precursor cells, thereby aggravating bone loss [23].

A number of studies confirmed that activation of the Keap1-Nrf2 axis is an important mechanism for cells to clear ROS [12, 42, 43]. However, the upstream signal molecules regulating the Keap1-Nrf2 axis in osteoclasts remain unknown. Our study found that Irgm1, as a protective protein of Keap1, adds an extra layer of regulation to the Keap1-Nrf2 axis. During RANKL-mediated osteoclast activation, the expressions of both Irgm1 and Keap1 were significantly increased. We verified that the IRGM-D2 domain bound directly or indirectly to KEAP1 and reduced the ubiquitinated species of KEAP1 to maintain its stability. Usp25 acts as a deubiquitinase for Keap1, and its binding to Keap1 is regulated by Irgm1 [12]. Irgm1 promoted Usp25-induced deubiquitination of Keap1 and prevented Keap1 from excessive ubiquitination and degradation. This process makes the cysteine residues of Keap1 less susceptible to oxidation, resulting in Nrf2 being unable to be released from Keap1 and then being degraded [15, 36]. The lack of Nrf2 caused the downstream antioxidant pathways to fail to activate normally, leading to the level of various antioxidants (Sod1, Sod2, Gpx, Cat, and Gr) remaining at low levels [14]. As a result, the excess ROS generated during RANKL-mediated osteoclastogenesis cannot be effectively eliminated. However, the loss of Irgm1 seems to help Nrf2 dissociate from Keap1 and maintain the stability of Nrf2 [44]. Based on this, we deduced that a small amount of Irgm1 in cells protected the activity of Keap1 from being ubiquitinated by other factors, including CUL3 or TRIM25 [12]. Indeed, we found that suppression of the proteasome pathway using MG132 reversed Keap1 degradation mediated by Irgm1 deletion. In addition, Irgm1 can bidirectionally regulate the half-life of Keap1.

In recent years, the crosstalk between ROS and NF-κB signaling pathways has been extensively studied [8]. NF-κB signaling was considered a key downstream factor of RANKL-mediated ROS signaling [4]. Consistent with our results, RANKL-mediated ROS activated the NF-κB pathway, inducing increased nuclear translocation of P65 [45, 46]. Moreover, loss of Irgm1 inhibited the phosphorylation of IκBα and P65, as well as the translocation of P65 into the nucleus, indicating that Irgm1 deletion reduced osteoclast differentiation and maturation via suppressing the NF-κB signal pathway.

Since Irgm1 showed excellent therapeutic effects on OVX-mediated osteoporosis in this study, it was considered a target to screen for specifically targeting drugs. Network medicine, as a new strategy in the field of drug discovery, uses network topology methods to infer the potential effects of drugs for in silico screening, and uses known drug-disease or drug-target associations to predict unknown associations [47]. In our study, mifamurtide attenuated bone loss induced by estrogen deficiency. As an effective inhibitor of Irgm1 predicted by the network medicine, mifamurtide activated the Keap1-Nrf2 signaling pathway and the expression of antioxidant enzymes at all doses [48]. Although mifamurtide exhibited significant therapeutic effects on osteoporosis, its direct binding to Irgm1 was only based on computational predictions. Experimental verification is needed in future studies to obtain safer and more effective dosages. As an immunomodulatory drug, mifamurtide has been proven to have clinical prospects in the treatment of osteosarcoma without serious side effects [49]. Recent studies have shown that mifamurtide maintains the balance of bone remodeling by regulating the balance of macrophage M1/M2 ratio [50]. Therefore, our study provided new insights into the role of mifamurtide in alleviating OVX-induced osteoporosis.

In this study, using double calcein labeling, we confirmed that the alleviation of OVX-mediated bone loss by Irgm1 deficiency was not only due to the inhibition of osteoclast bone resorption, but also closely related to the enhancement of osteoblast bone formation. We believed that the secretion of macrophage cytokines may be affected to a certain extent after specific knockout of Irgm1 in cells of the myeloid lineage [51]. Notably, Irgm1 haplodeficiency reduced the percentage of M1 phenotype macrophages but showed no effect on M2. Our results demonstrated that Irgm1-deficient RAW264.7 cells showed less expression of CD86, an M1-polarized phenotype. The decrease of the M1/M2 ratio is beneficial to the osteogenic function of BMSCs [52, 53]. In addition, M1 macrophages can promote bone resorption through multiple mechanisms, including serving as an osteoclast reservoir and secreting pro-inflammatory cytokines [37]. This may also be an important reason why Irgm1 deficiency inhibits osteoclast formation. In future studies, we will further clarify the regulatory mechanism of Irgm1 deficiency on macrophage polarization and its impact on osteogenic differentiation, so as to improve the regulatory mechanism of Irgm1 on bone metabolism.

## Conclusion

In conclusion, our study confirmed that Irgm1 hindered the defense function of the antioxidant system by maintaining the protein level of Keap1 (Fig. 8M), causing intracellular ROS accumulation and then activating the downstream NF-κB signal pathway to lead to osteoclastogenesis. The Irgm1 inhibitor mifamurtide can alleviate bone loss in mice, suggesting that mifamurtide could be developed as a therapeutic agent for postmenopausal osteoporosis. Since the antioxidant function of the Keap1-Nrf2 axis is beneficial to many diseases, our discovery has opened up new paths for the exploration and application of Irgm1 inhibitors in the treatment of various diseases in the future.

## Supplementary information

Supplememt data

Original Western blots

## Data Availability

The data used in the study to support the main findings will be available from the corresponding author upon request.
