# Supplementary material for: Targeting Irgm1 to combat osteoporosis: suppressing ROS and restoring bone remodeling
Source: Cell Death Dis. 2025 Aug 27;16(1):651. doi: 10.1038/s41419-025-07965-7 (PMC12391319; doi:10.1038/s41419-025-07965-7)
Supplement: Supplementary file 1 — Supplememt data [file 41419_2025_7965_MOESM1_ESM.docx]

**Supplement Data**


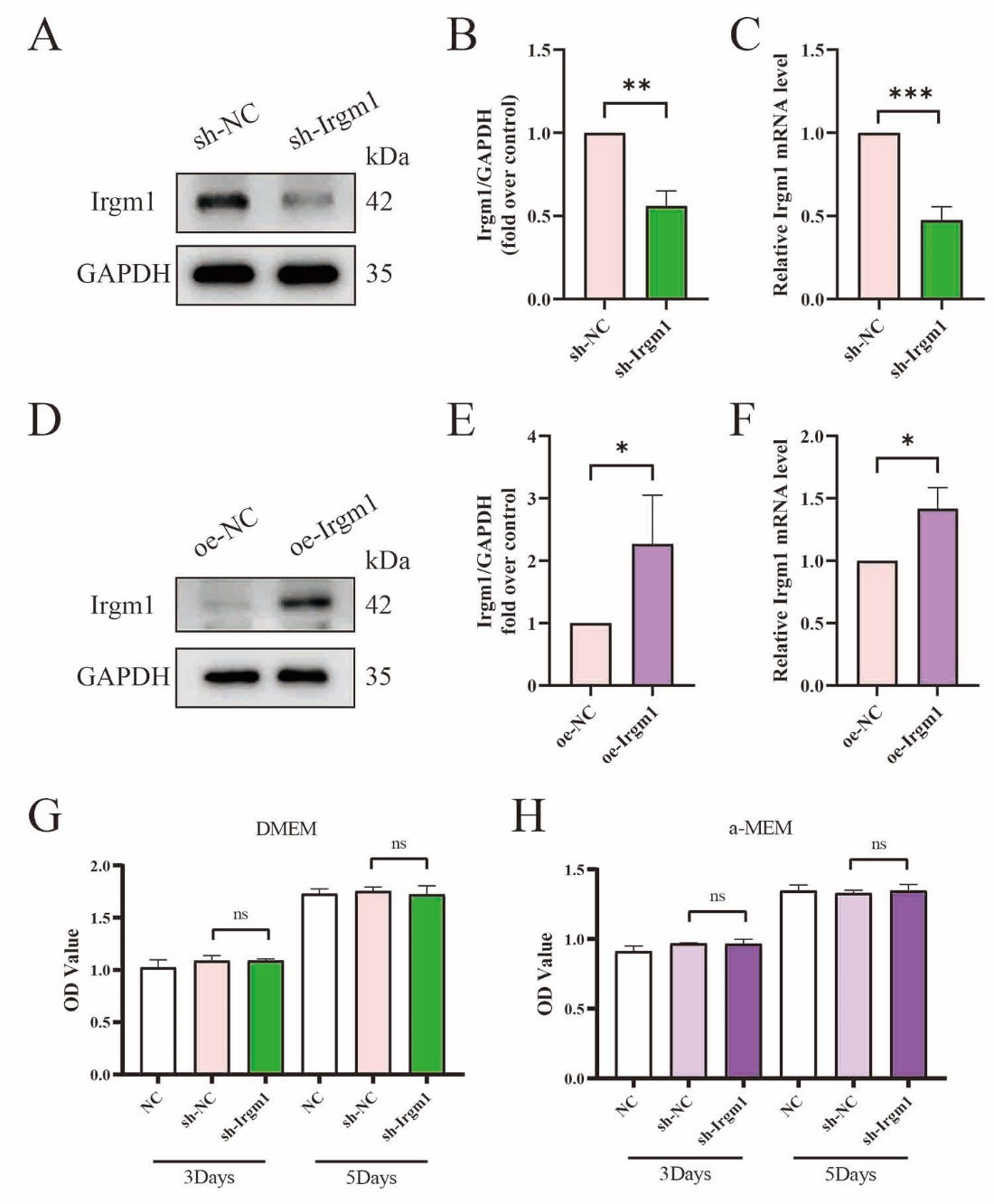


**Supplement Figure 1. Negative control and Irgm1 knockdown lentivirus show no effect on RAW264.7 cell viability. (A)** WB analysis of the efficiency of Irgm1 knockdown lentivirus transfection (sh-Irgm1). **(B)** Quantitative analysis of data from **(A)** (n = 3). ∗∗*p* < 0.01 VS the indicated groups. **(C)** RT-qPCR showed Irgm1 knockdown efficiency at mRNA level. Quantitative results were normalized to GAPDH and presented as means ± SD (n = 3), ∗∗∗*p* < 0.001 VS the indicated groups. **(D)** WB analysis of the efficiency of Irgm1 overexpression lentivirus transfection (oe-Irgm1). **(E)** Quantitative analysis of data from **(D)** (n = 3). ∗*p* < 0.05 VS the indicated groups. **(F)** Irgm1 mRNA levels after overexpression lentivirus transfection. Quantitative results were normalized to GAPDH and presented as means ± SD (n = 3), ∗*p* < 0.05 VS the indicated groups. **(G-H)** CCK-8 assays for RAW264.7 cells viability test. Cells were cultured in DMEM **(G)** or α-MEM **(H)** containing 10% FBS for indicated times (n=3), ns VS the indicated groups.

**Supplement table 1. The primers used for Irgm1 conditional knockout mice genotyping.**

| **Primer** | **Sequence (5'-3')** |
| --- | --- |
| Irgm1-Flox | F: CAGTGAAGACTGACCTTCGCA |
|  | R: ACCACCTAAGCAAGGATGCC |
| Lyz2-Cre | F: AAGGAGGGACTTGGAGGATG |
|  | R: ACCGGTAATGCAGGCAAAT |

**Supplement table 2. The primer sequences used for RT-qPCR.**

| **Gene Symbol (GenBank Accession No.)** | **Sequence (5'-3')** |
| --- | --- |
| Irgm1 (NM_008326.2) | F:TGCTCCACTACTCCCCAACAT |
|  | R:GCTCCTACTGACCTCAGGTAAC |
| Keap1 (NM_001110307.1) | F:CAGCTACACACTAGAGGATCACA |
|  | R:GTGGATGCCTTCGATGGACA |
| Nrf2 (NM_010902.5) | F:CTGAACTCCTGGACGGGACTA |
|  | R:CGGTGGGTCTCCGTAAATGG |
| Traf6 (NM_001303273.1) | F: AAAGCGAGAGATTCTTTCCCTG |
|  | R: ACTGGGGACAATTCACTAGAGC |
| Nfatc1 (NM_001164112.1) | F: CCGTTGCTTCCAGAAAATAACA |
|  | R: TGTGGGATGTGAACTCGGAA |
| Ctsk (NM_007802.4) | F: CTTCCAATACGTGCAGCAGA |
|  | R: TCTTCAGGGCTTTCTCGTTC |
| Sod1 (NM_011434.2) | F: AACCAGTTGTGTTGTCAGGAC |
|  | R: CCACCATGTTTCTTAGAGTGAGG |
| Sod2 (NM_013671.3) | F: TGGACAAACCTGAGCCCTAAG |
|  | R: CCCAAAGTCACGCTTGATAGC |
| Cat (NM_009804.2) | F: GGAGTCTTCGTCCCGAGTCT |
|  | R: CGGTCTTGTAATGGAACTTGC |
| Gpx (NM_001329527.1) | F: AGTCCACCGTGTATGCCTTCT |
|  | R: GAGACGCGACATTCTCAATGA |
| Gr (NM_010344.4) | F: GCGTGAATGTTGGATGTGTACC |
|  | R: GTTGCATAGCCGTGGATAATTTC |
| GAPDH (NM_001411843.1) | F: TGACCACAGTCCATGCCATC |
|  | R: GACGGACACATTGGGGGTAG |

**Supplement table 3. 82 common up-regulated targets of RANKL-mediated osteoclastogenesis for days 1, 2, and 3 from GSE176265 dataset.**

| **Number** | **Protein name** | **Gene name** |
| --- | --- | --- |
| 1 | Adhesion G protein-coupled receptor E1 | Adgre1 |
| 2 | Adenylosuccinate synthetase, non muscle | Adss |
| 3 | Expressed sequence AI607873 | AI607873 |
| 4 | Allograft inflammatory factor 1 | Aif1 |
| 5 | Caveolin 2 | Cav2 |
| 6 | Chemokine (C-C motif) ligand 5 | Ccl5 |
| 7 | Cyclin L1 | Ccnl1 |
| 8 | CD72 antigen | Cd72 |
| 9 | Cold shock domain containing E1, RNA binding | Csde1 |
| 10 | Cathepsin A | Ctsa |
| 11 | Cathepsin K | Ctsk |
| 12 | Chemokine (C-X-C motif) ligand 16 | Cxcl16 |
| 13 | Cytochrome b5 reductase 3 | Cyb5r3 |
| 14 | Cytohesin 4 | Cyth4 |
| 15 | Aspartyl-tRNA synthetase | Dars |
| 16 | Dentrocyte expressed seven transmembrane protein | Dcstamp |
| 17 | DEAD (Asp-Glu-Ala-Asp) box polypeptide 5 | Ddx5 |
| 18 | DEAD (Asp-Glu-Ala-Asp) box polypeptide 58 | Ddx58 |
| 19 | Docking protein 1 | Dok1 |
| 20 | Eukaryotic translation elongation factor 2 | Eef2 |
| 21 | Family with sequence similarity 26, member F | Fam26f |
| 22 | Guanylate binding protein 2 | Gbp2 |
| 23 | Predicted gene 4951 | Gm4951 |
| 24 | G protein-coupled receptor 137B | Gpr137b |
| 25 | G protein-coupled receptor 68 | Gpr68 |
| 26 | Gasdermin D | Gsdmd |
| 27 | Histocompatibility 2, M region locus 2 | H2-M2 |
| 28 | Histocompatibility 2, T region locus 10 | H2-T10 |
| 29 | Heterogeneous nuclear ribonucleoprotein A2/B1 | Hnrnpa2b1 |
| 30 | Heterogeneous nuclear ribonucleoprotein K | Hnrnpk |
| 31 | Heat shock protein 8 | Hspa8 |
| 32 | Heat shock protein 1 (chaperonin) | Hspd1 |
| 33 | Interferon-induced protein with tetratricopeptide repeats 2 | Ifit2 |
| 34 | Interferon-induced protein with tetratricopeptide repeats 3 | Ifit3 |
| 35 | Interleukin 2 receptor, gamma chain | Il2rg |
| 36 | Interferon regulatory factor 1 | Irf1 |
| 37 | Interferon regulatory factor 7 | Irf7 |
| 38 | Immunity-related GTPase family M member 1 | Irgm1 |
| 39 | Influenza virus NS1A binding protein | Ivns1abp |
| 40 | LIM and senescent cell antigen like domains 2 | Lims2 |
| 41 | Lysophosphatidylcholine acyltransferase 2 | Lpcat2 |
| 42 | Megakaryocyte-associated tyrosine kinase | Matk |
| 43 | Matrix metallopeptidase 14 (membrane-inserted) | Mmp14 |
| 44 | Matrix metallopeptidase 9 | Mmp9 |
| 45 | Myeloid nuclear differentiation antigen like | Mndal |
| 46 | Nuclear receptor coactivator 4 | Ncoa4 |
| 47 | Nebulin-related anchoring protein | Nrap |
| 48 | 2'-5' oligoadenylate synthetase-like 2 | Oasl2 |
| 49 | Purinergic receptor P2Y, G-protein coupled, 14 | P2ry14 |
| 50 | Poly(A) binding protein, cytoplasmic 1 | Pabpc1 |
| 51 | PAP associated domain containing 4 | Papd4 |
| 52 | Phosphodiesterase 4D interacting protein (myomegalin) | Pde4dip |
| 53 | Platelet derived growth factor, B polypeptide | Pdgfb |
| 54 | Phospholipid transfer protein | Pltp |
| 55 | Phorbol-12-myristate-13-acetate-induced protein 1 | Pmaip1 |
| 56 | PTPRF interacting protein, binding protein 2 (liprin beta 2) | Ppfibp2 |
| 57 | Proteasome (prosome, macropain) 26S subunit, non-ATPase, 10 | Psmd10 |
| 58 | Protein tyrosine phosphatase, non-receptor type 12 | Ptpn12 |
| 59 | Protein tyrosine phosphatase, receptor type, O | Ptpro |
| 60 | RAS p21 protein activator 4 | Rala |
| 61 | Radical S-adenosyl methionine domain containing 2 | Rasa4 |
| 62 | Scinderin | Rnf114 |
| 63 | SH3-domain kinase binding protein 1 | Rsad2 |
| 64 | Solute carrier family 29 (nucleoside transporters), member 1 | Scin |
| 65 | Signal recognition particle 54A | Sema4d |
| 66 | TAP binding protein | Sh3kbp1 |
| 67 | T cell, immune regulator 1, ATPase, H+ transporting, lysosomal V0 protein A3 | Shtn1 |
| 68 | TNF receptor-associated factor 1 | Slc25a36 |
| 69 | Tripartite motif-containing 30D | Slc29a1 |
| 70 | V-ral simian leukemia viral oncogene A (ras related) | Slc9b2 |
| 71 | Ring finger protein 114 | Src |
| 72 | Sema domain, immunoglobulin domain (Ig), transmembrane domain (TM) and short cytoplasmic domain, (semaphorin) 4D | Srp54a |
| 73 | Shootin 1 | Srsf5 |
| 74 | Solute carrier family 25, member 36 | Tapbp |
| 75 | Solute carrier family 9, subfamily B (NHA2, cation proton antiporter 2), member 2 | Tcirg1 |
| 76 | Rous sarcoma oncogene | Tmem176b |
| 77 | Serine/arginine-rich splicing factor 5 | Tmem204 |
| 78 | Transmembrane protein 176B | Tor3a |
| 79 | Transmembrane protein 204 | Traf1 |
| 80 | Torsin family 3, member A | Trafd1 |
| 81 | TRAF type zinc finger domain containing 1 | Trim30d |
| 82 | Tripartite motif-containing 34A | Trim34a |

**Supplement table 4. 11 IRGM-related genes from STRING database.**

| **Number** | **Protein name** | **Gene name** |
| --- | --- | --- |
| 1 | Zinc finger protein 300 | ZNF300 |
| 2 | Tetraspanin 8 | TSPAN8 |
| 3 | SNAP associated protein | SNAPIN |
| 4 | SH3 domain containing GRB2 like, endophilin B1 | SH3GLB1 |
| 5 | Autophagy related 10 | ATG10 |
| 6 | Autophagy related 16 like 1 | ATG16L1 |
| 7 | Autophagy related 5 | ATG5 |
| 8 | Beclin 1 | BECN1 |
| 9 | DExD/H-box helicase 58 | DDX58 |
| 10 | Immunity related GTPase M | IRGM |
| 11 | Nucleotide binding oligomerization domain containing 2 | NOD2 |

**Supplement table 5. 17 candidate drugs related to the IRGM-related gene sets.**

| **Drug_id** | **Drug_name** | **Distances** | **P value** |
| --- | --- | --- | --- |
| DB13615 | Mifamurtide | -2.246530722 | 6.93E-30 |
| DB00783 | Estradiol | 0.863822381 | 1.12E-10 |
| DB13952 | Estradiol acetate | 1.204675667 | 1.34E-07 |
| DB13953 | Estradiol benzoate | 1.204675667 | 1.34E-07 |
| DB13954 | Estradiol cypionate | 1.204675667 | 1.34E-07 |
| DB13955 | Estradiol dienanthate | 1.204675667 | 1.34E-07 |
| DB13956 | Estradiol valerate | 1.204675667 | 1.34E-07 |
| DB01017 | Minocycline | 1.368421053 | 9.86E-11 |
| DB00945 | Acetylsalicylic acid | 1.37037037 | 3.25E-14 |
| DB04315 | Guanosine-5'-Diphosphate | 1.742857143 | 1.55E-07 |
| DB12010 | Fostamatinib | 1.840740741 | 1.96E-30 |
| DB11638 | Artenimol | 1.853333333 | 3.36E-09 |
| DB09130 | Copper | 1.872180451 | 1.31E-14 |
| DB14533 | Zinc chloride | 1.934065934 | 6.08E-08 |
| DB14548 | Zinc sulfate, unspecified form | 1.934065934 | 6.08E-08 |
| DB01593 | Zinc | 1.946428571 | 1.39E-08 |
| DB14487 | Zinc acetate | 1.946428571 | 1.39E-08 |
