# Supplementary material for: Targeting Irgm1 to combat osteoporosis: suppressing ROS and restoring bone remodeling
Source: Cell Death Dis. 2025 Aug 27;16(1):651. doi: 10.1038/s41419-025-07965-7 (PMC12391319; doi:10.1038/s41419-025-07965-7)

Figure 1A

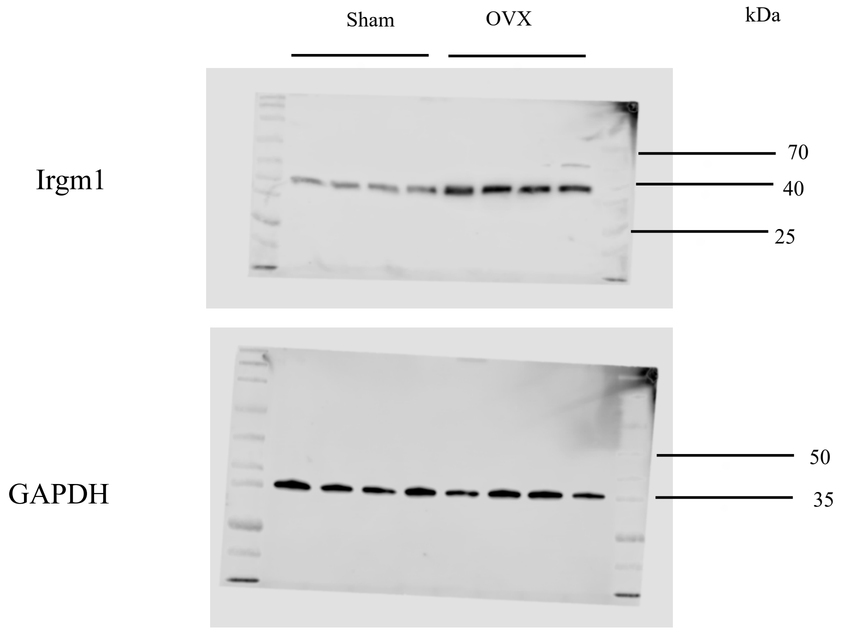

Figure 1H

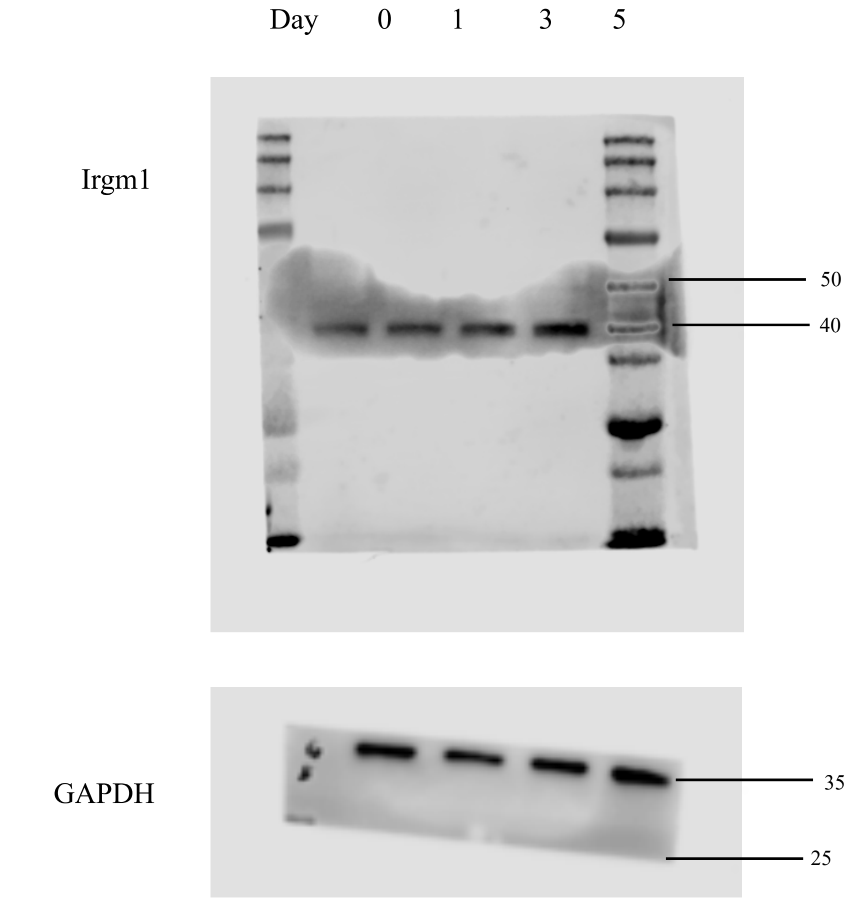

Figure 1I

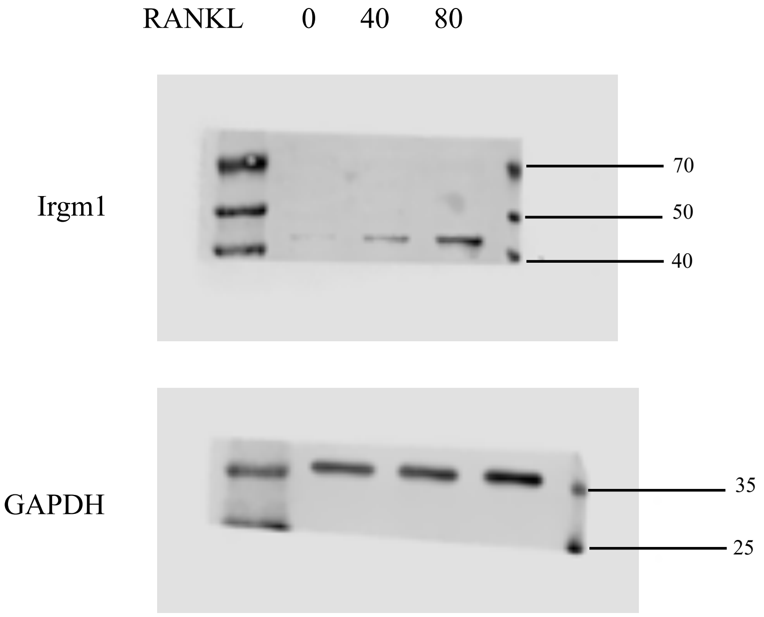

Figure 2C

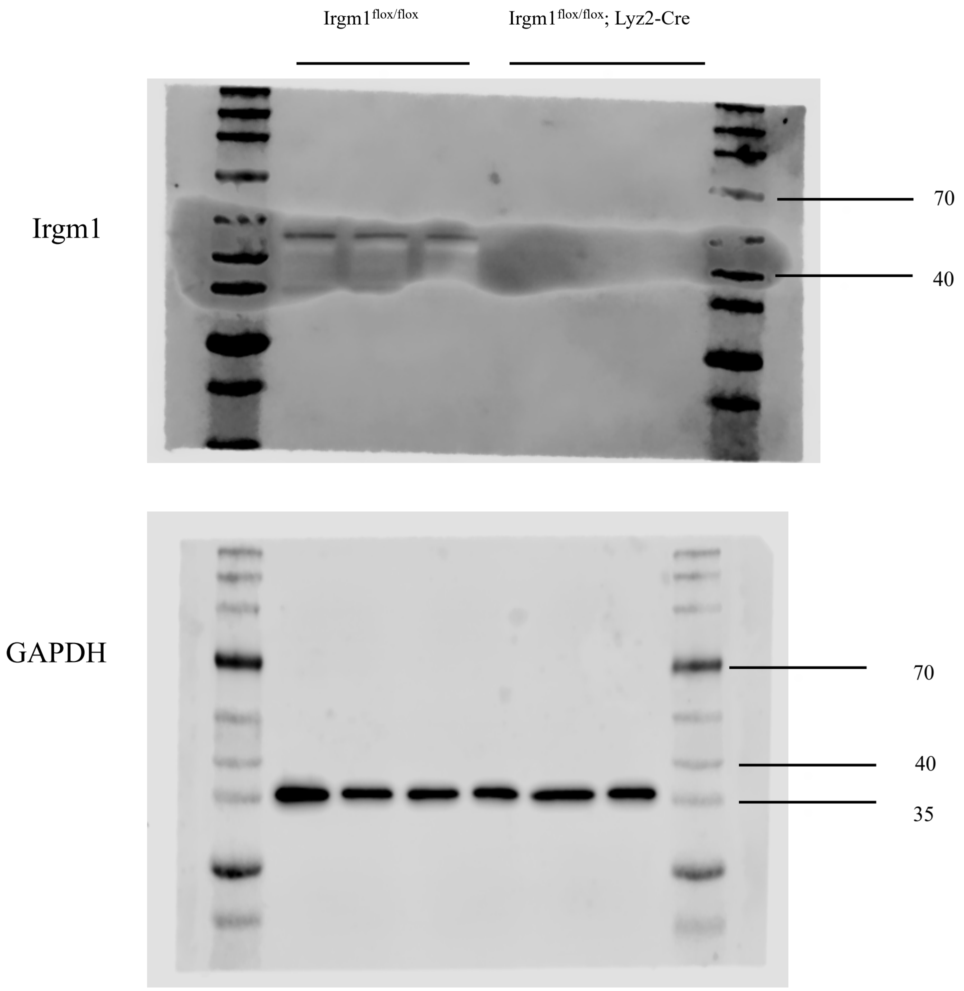

Irgm1$^{flox/flox}$
Irgm1$^{flox/flox}$; Lyz2-Cre
Irgm1
70
40
GAPDH
70
40
35

Figure 4A

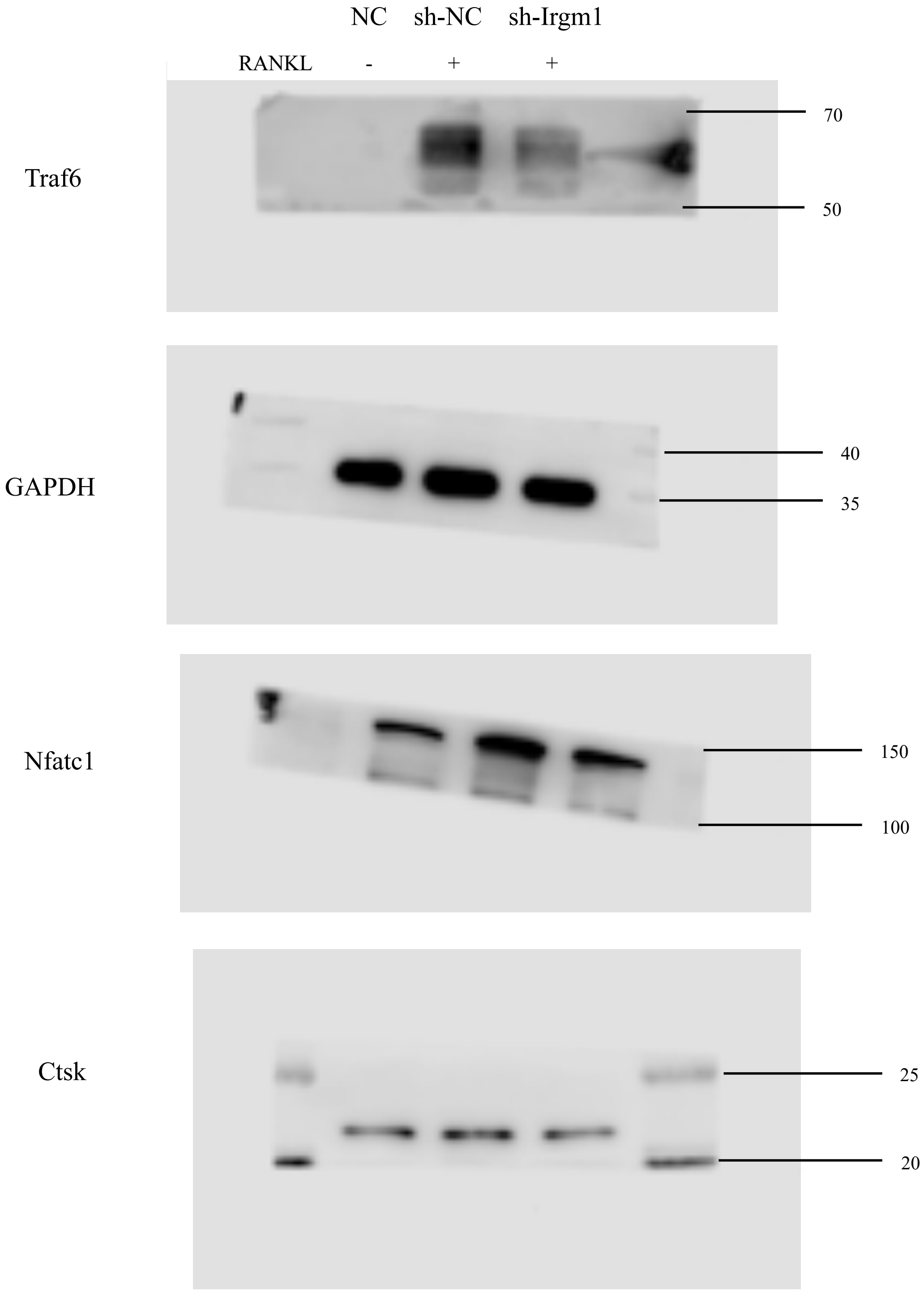
NC
sh-NC
sh-Irgm1
RANKL
-
+
+
Traf6
70
50
GAPDH
40
35
Nfatc1
150
100
Ctsk
25
20

Figure 4P

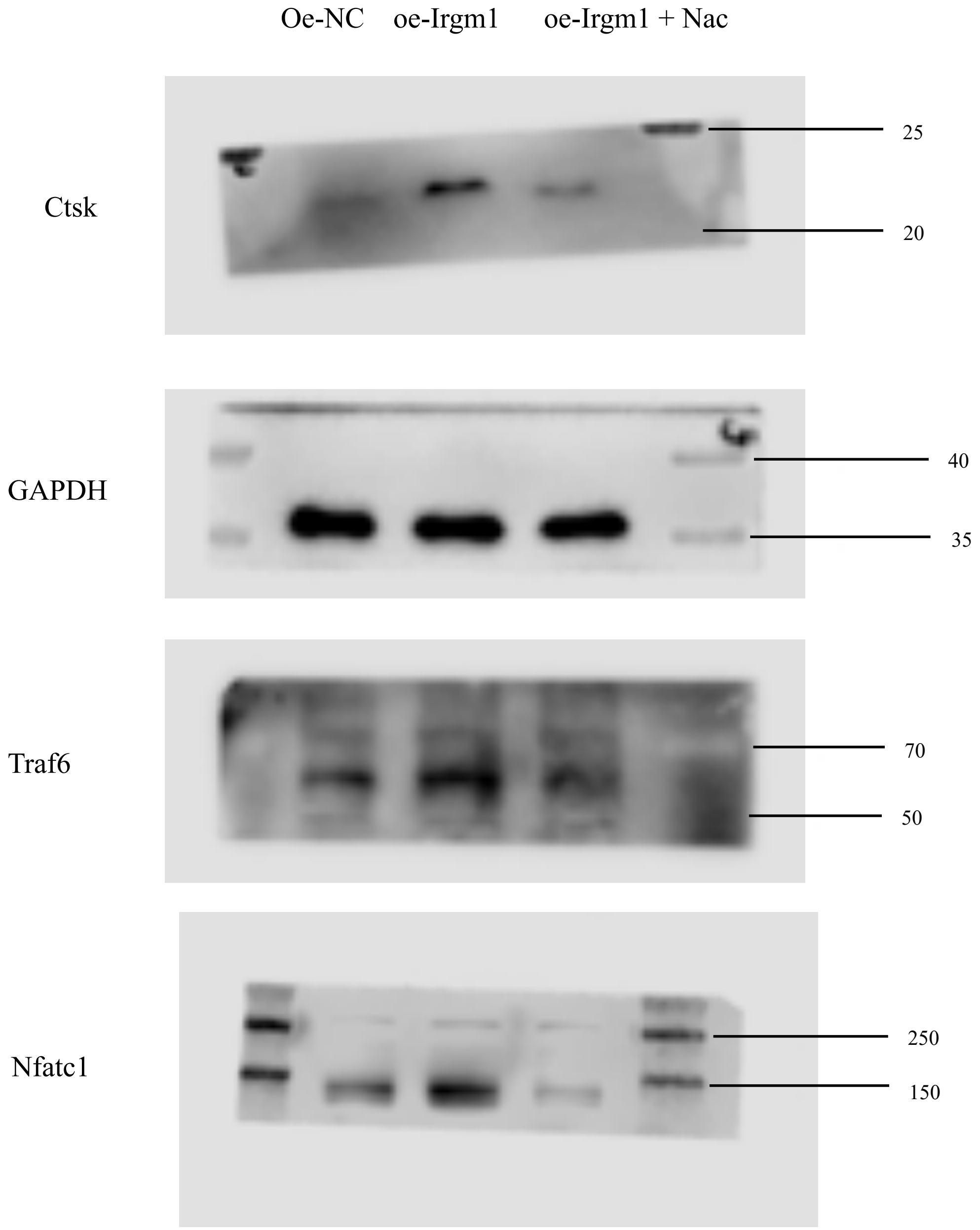
Oe-NC
oe-Irgm1
oe-Irgm1 + Nac
Ctsk
25
20
GAPDH
40
35
Traf6
70
50
Nfatc1
250
150

Figure 5A

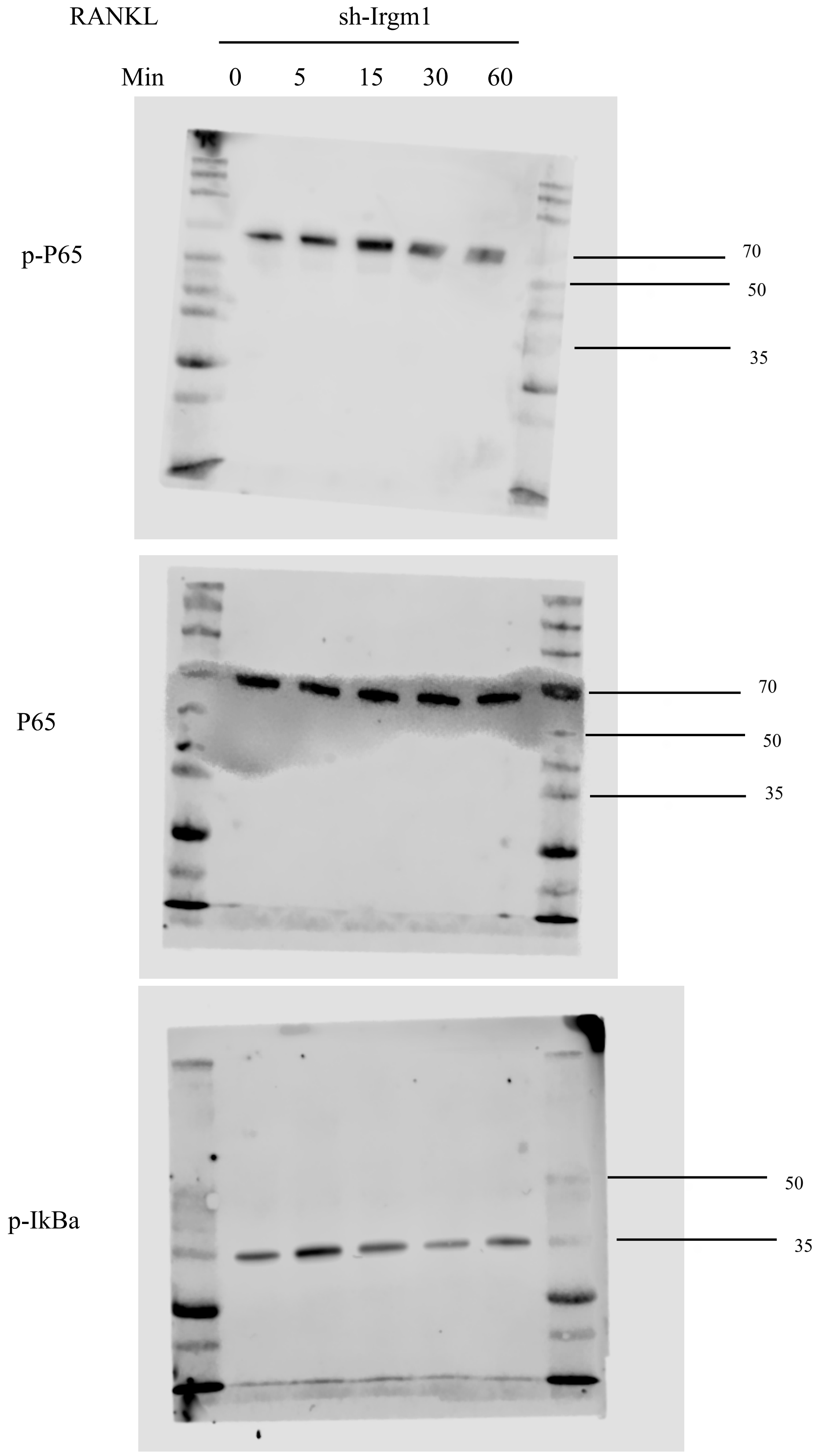
RANKL
sh-Irgm1
Min
0
5
15
30
60
p-P65
70
50
35
P65
70
50
35
p-IkBa
50
35

IkBa

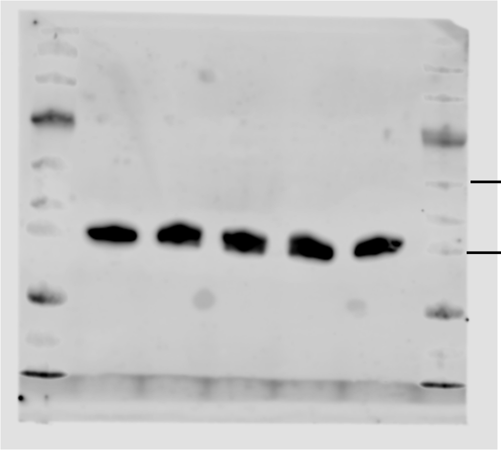

50

35

GAPDH

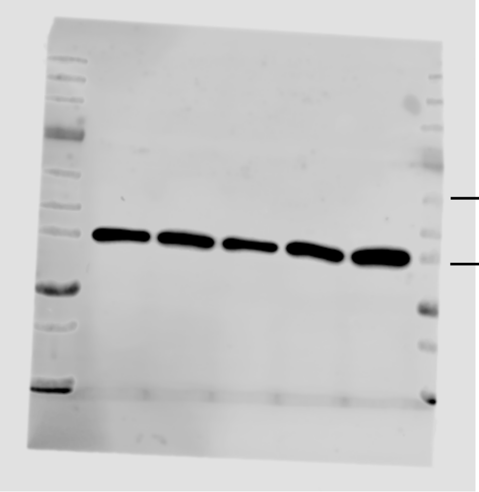

50

35

RANKL sh-NC

Min 0 5 15 30 60

p-P65

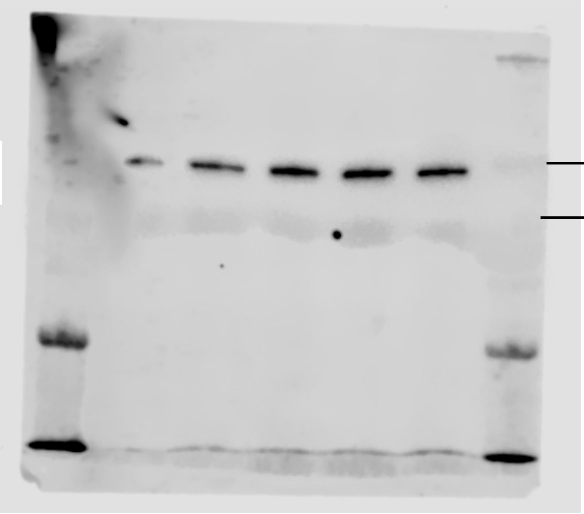

70

50

P65

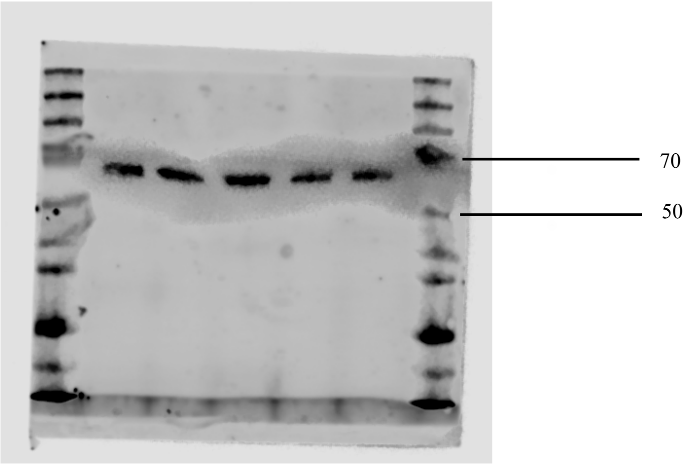

70

50

p-IkBa

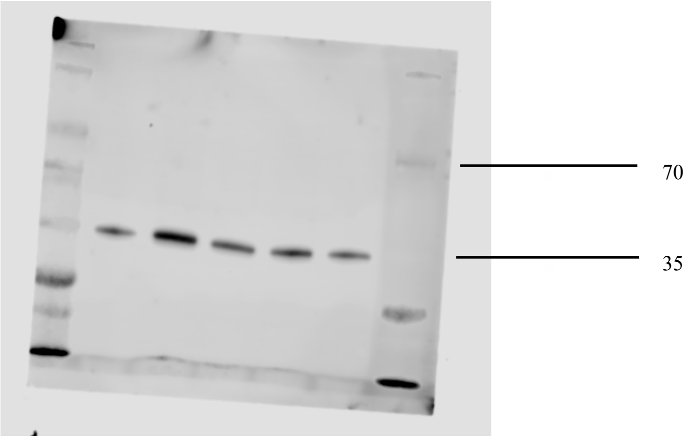

70

35

IkBa

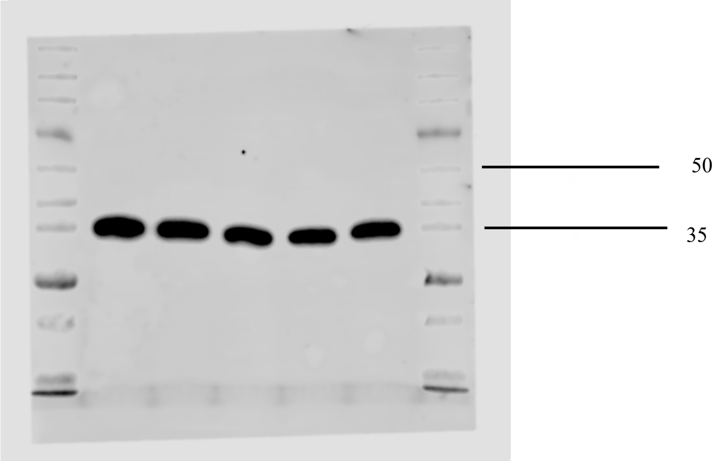

50

35

GAPDH

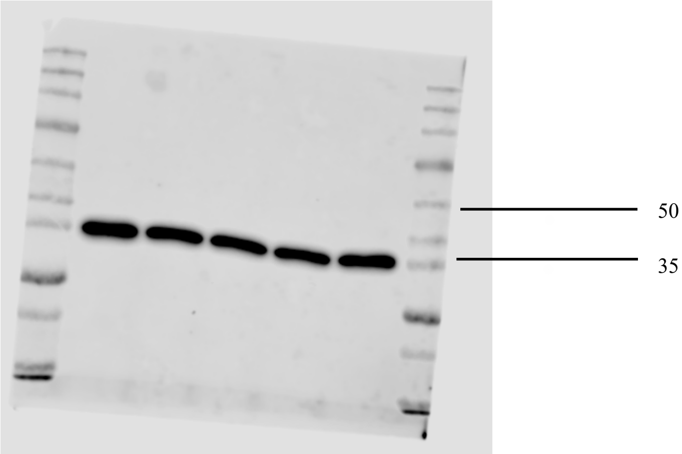

50

35

Figure 6B

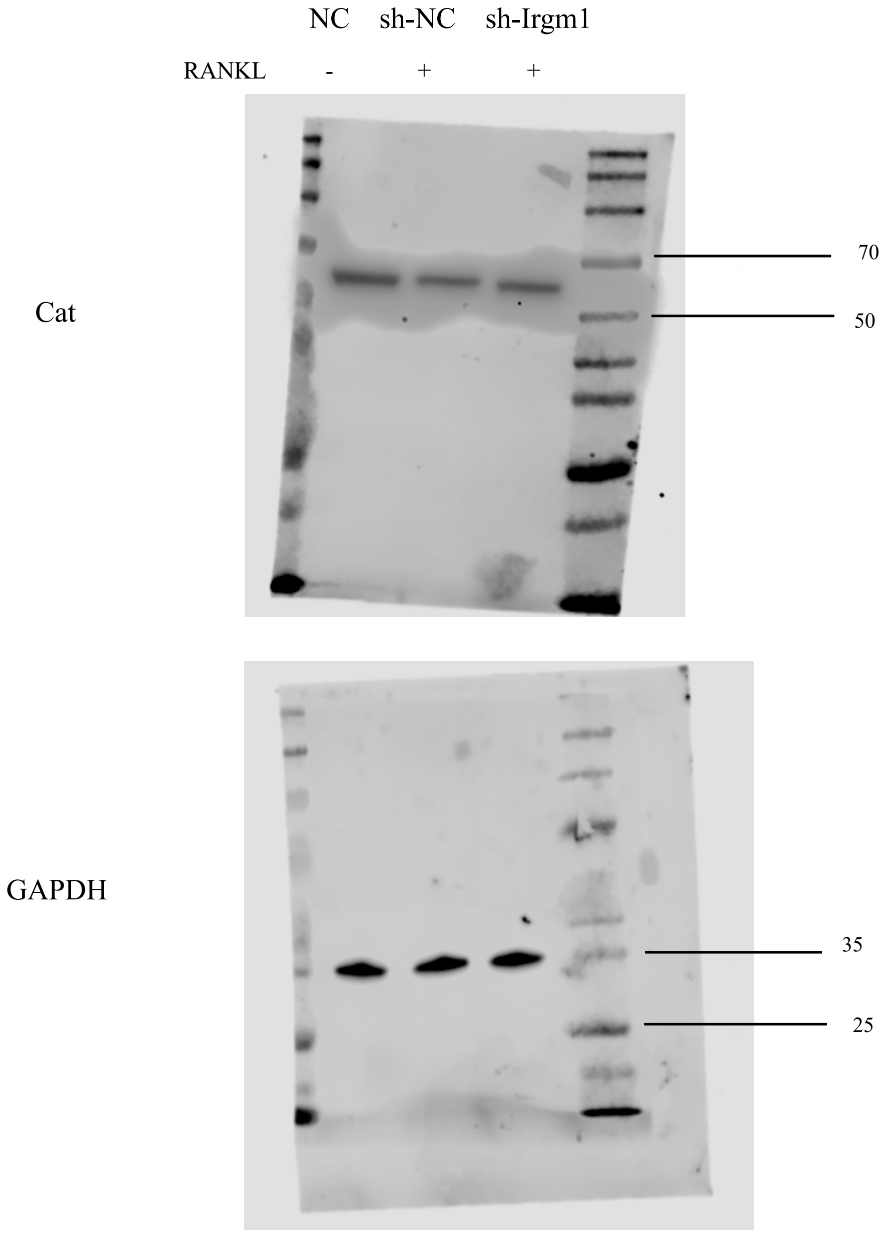

Figure 6C

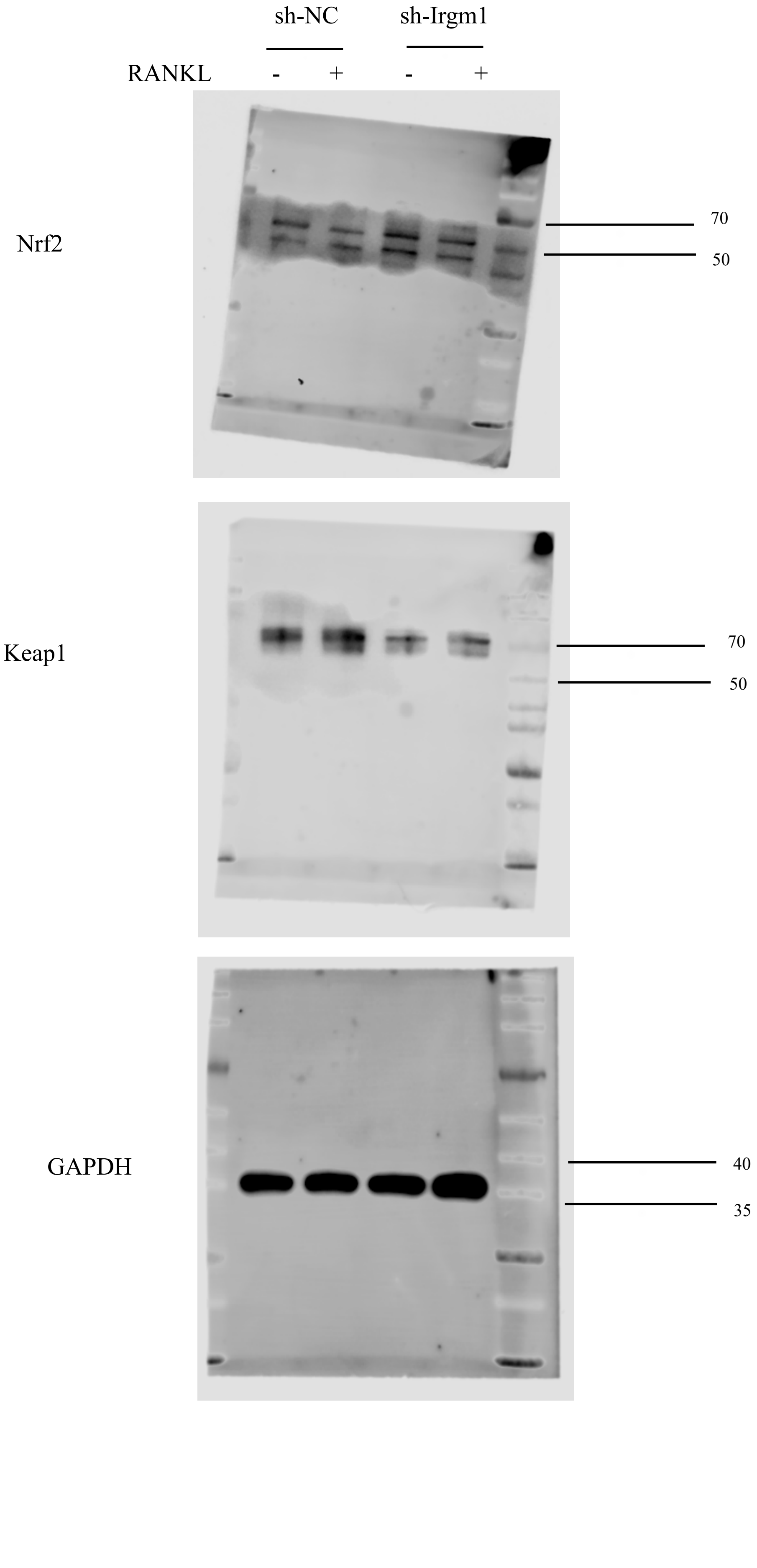

Figure 6H

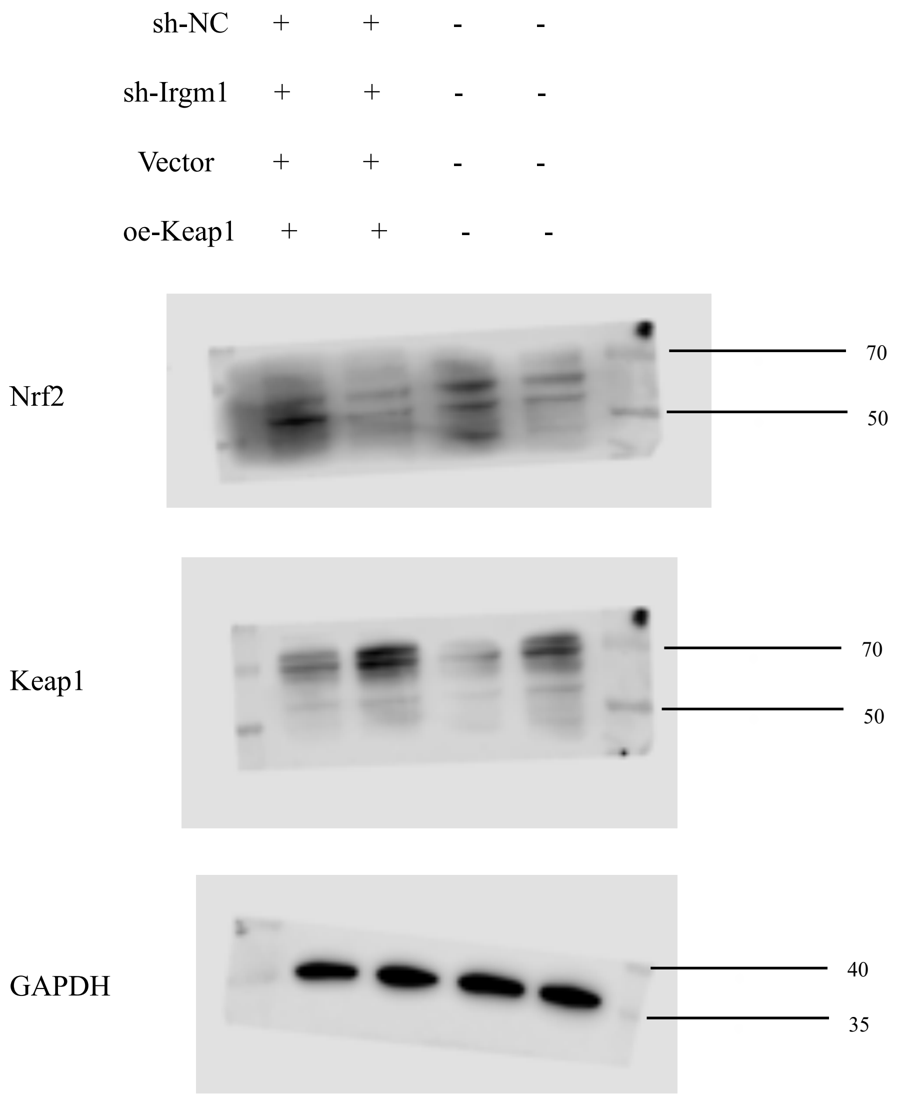
sh-NC + + - -
sh-Irgm1 + + - -
Vector + + - -
oe-Keap1 + + - -
Nrf2
Keap1
GAPDH
70
50
70
50
40
35

Figure 7B

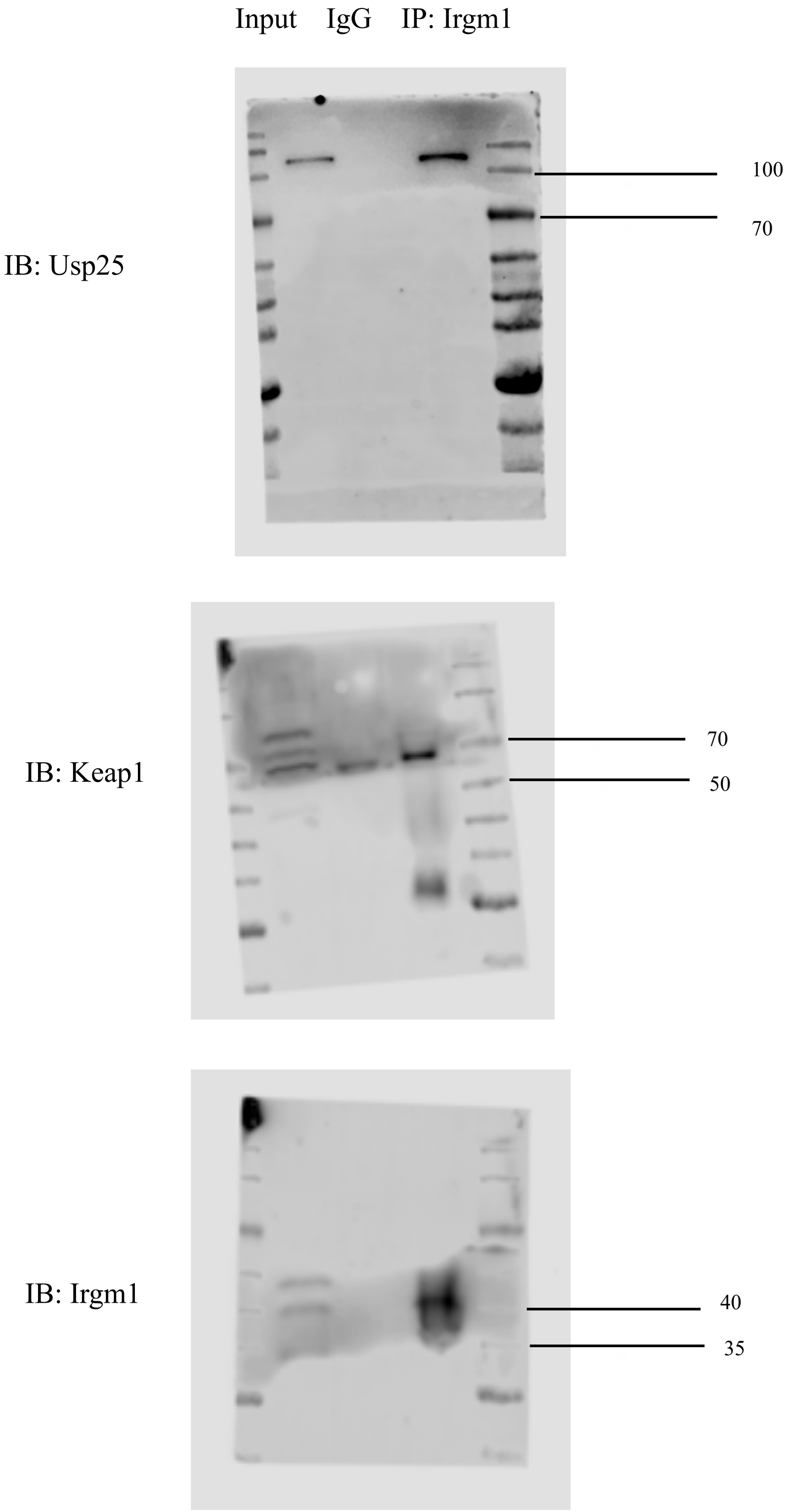

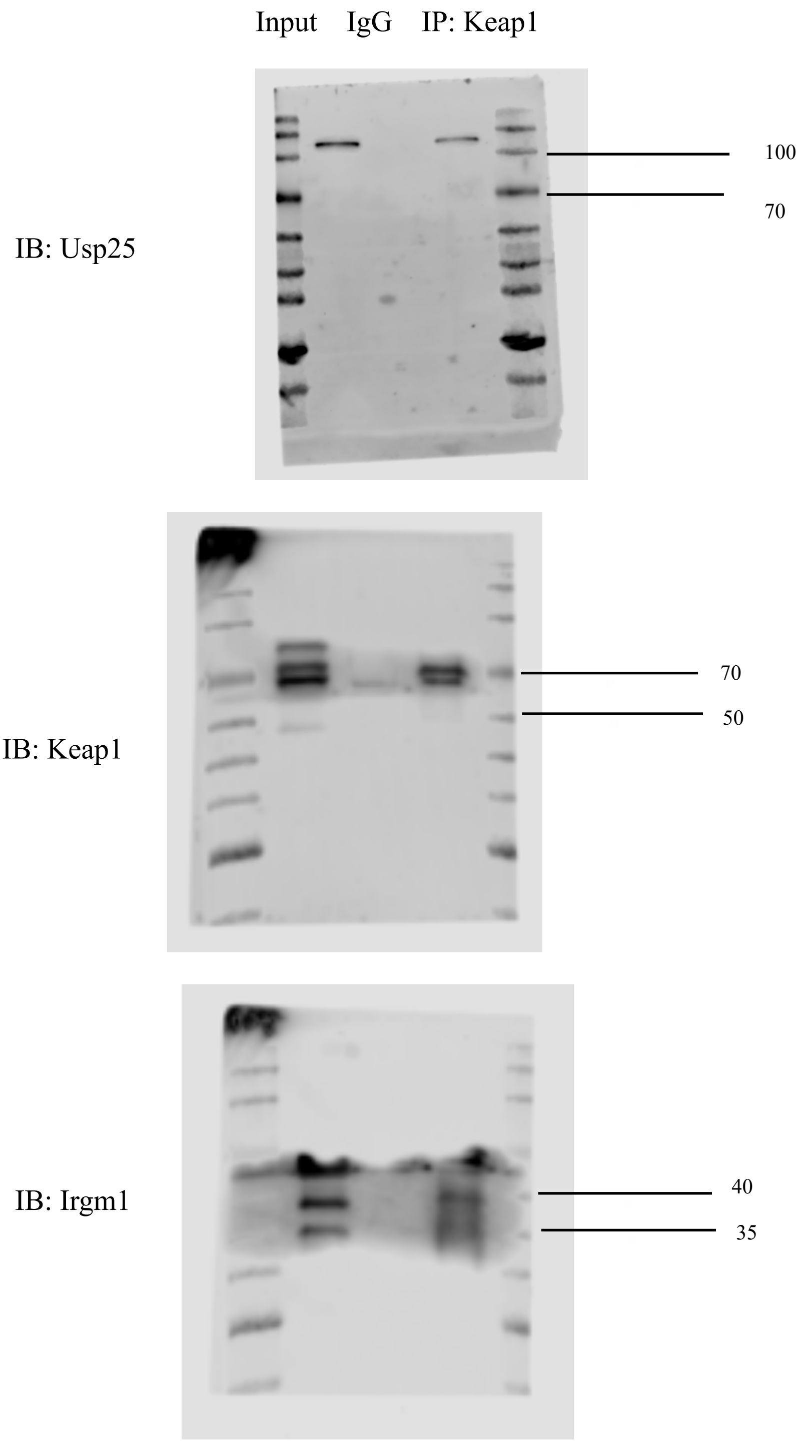
Input
IgG
IP: Keap1
IB: Usp25
100
70
IB: Keap1
70
50
IB: Irgm1
40
35

Figure 7C

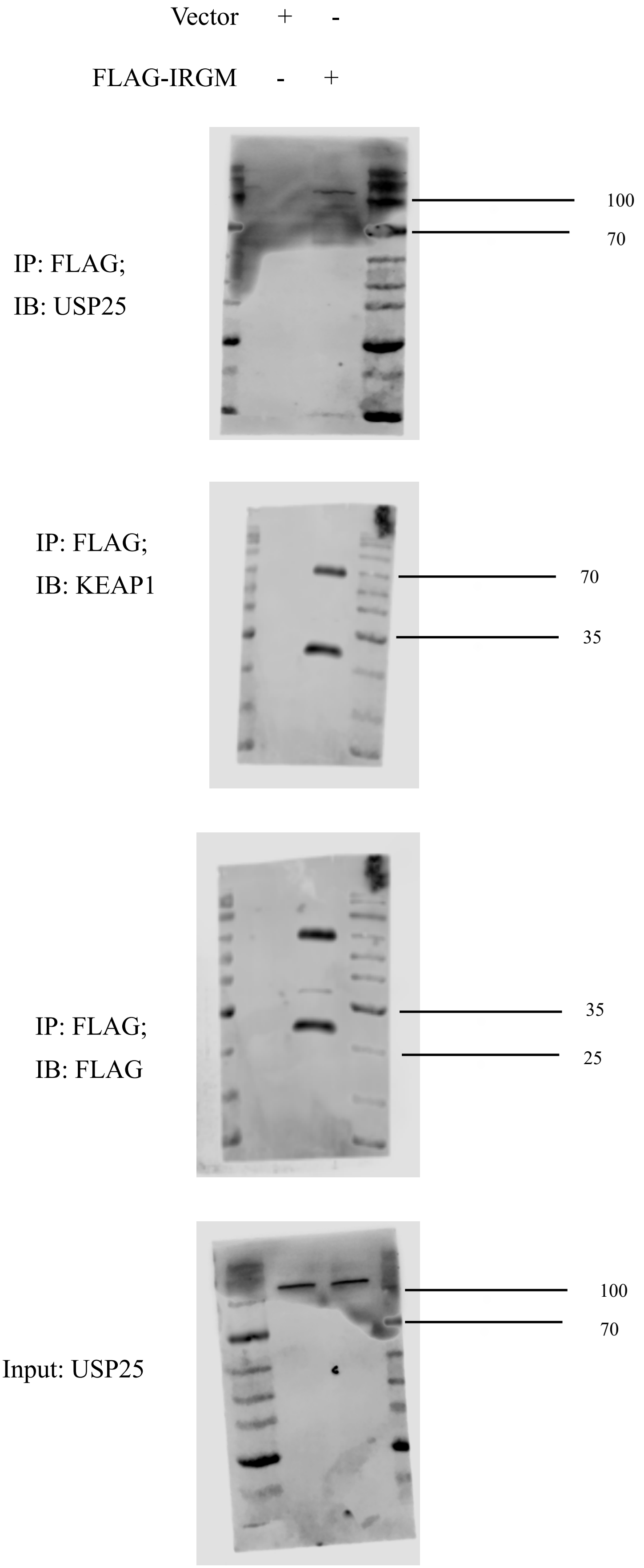

Vector + -
FLAG-IRGM - +
IP: FLAG;
IB: USP25
100
70
IP: FLAG;
IB: KEAP1
70
35
IP: FLAG;
IB: FLAG
35
25
Input: USP25
100
70

Input: KEAP1

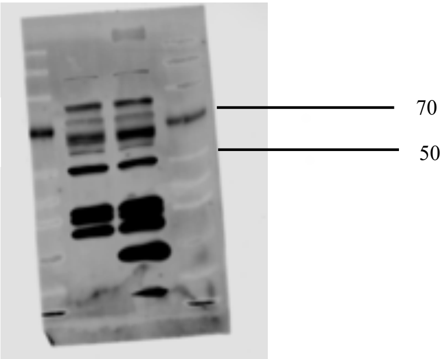

Input: FLAG

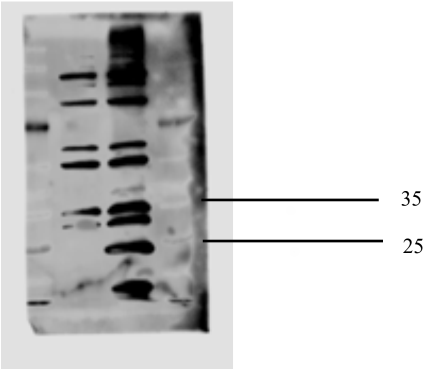

Input: GAPDH

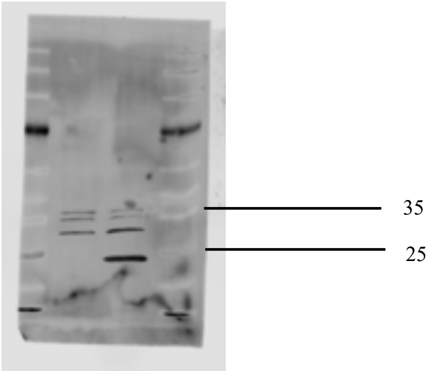

Vector	+	-
HA-KEAP1	-	+

IP: HA;

IB: USP25

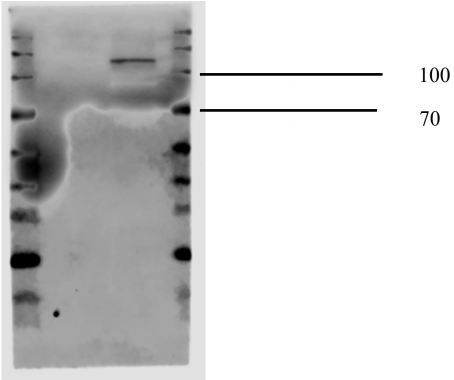

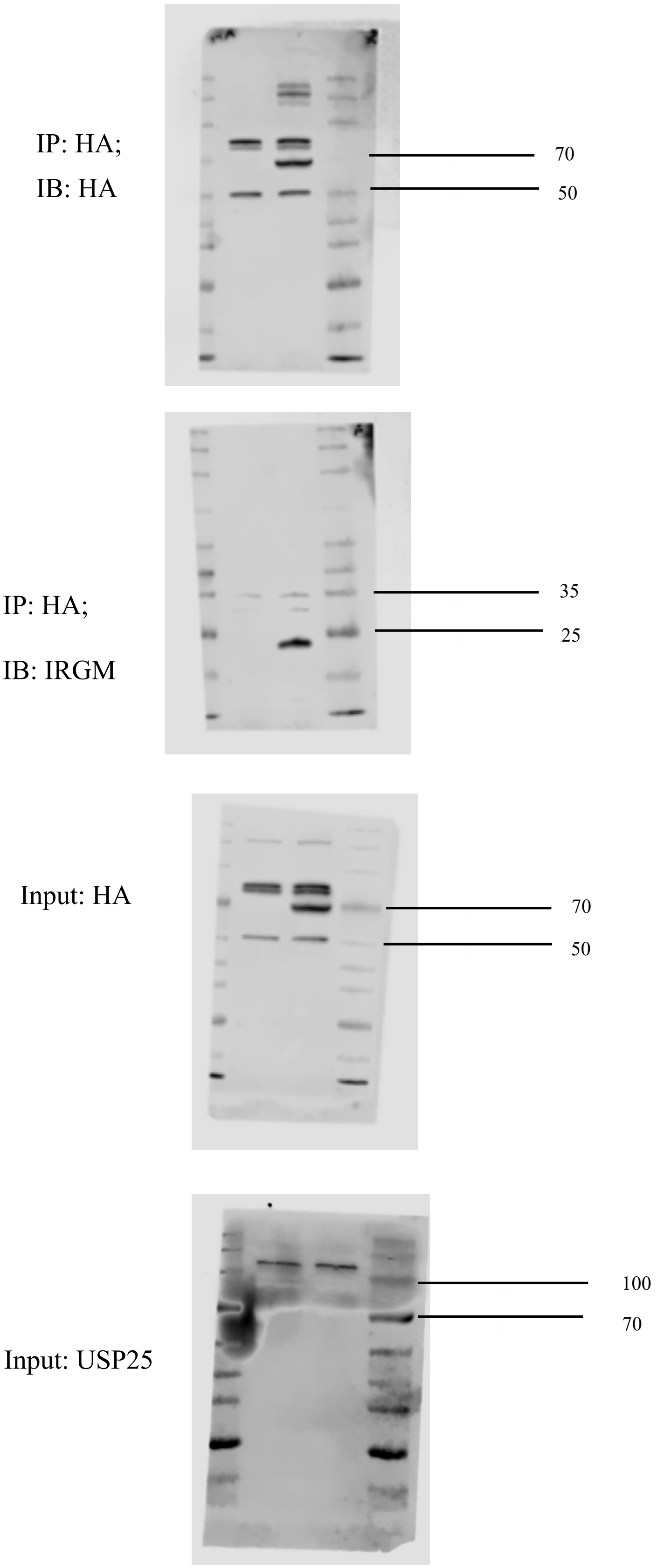
IP: HA;
IB: HA
70
50
IP: HA;
IB: IRGM
35
25
Input: HA
70
50
Input: USP25
100
70

Input: IRGM

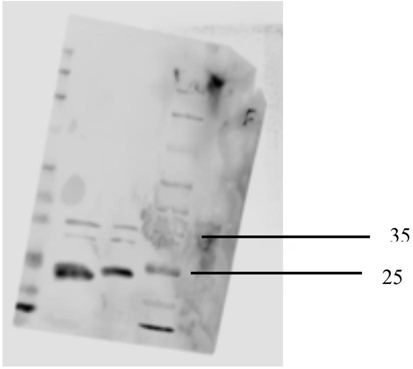

Input: GAPDH

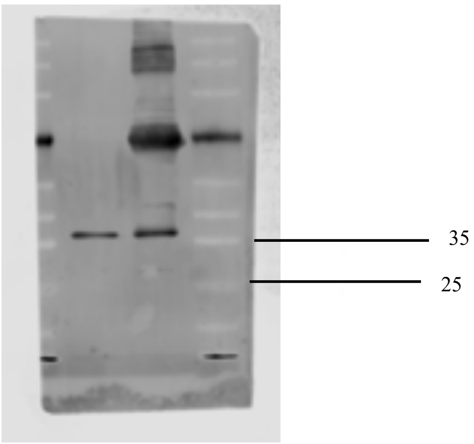

Figure 7D

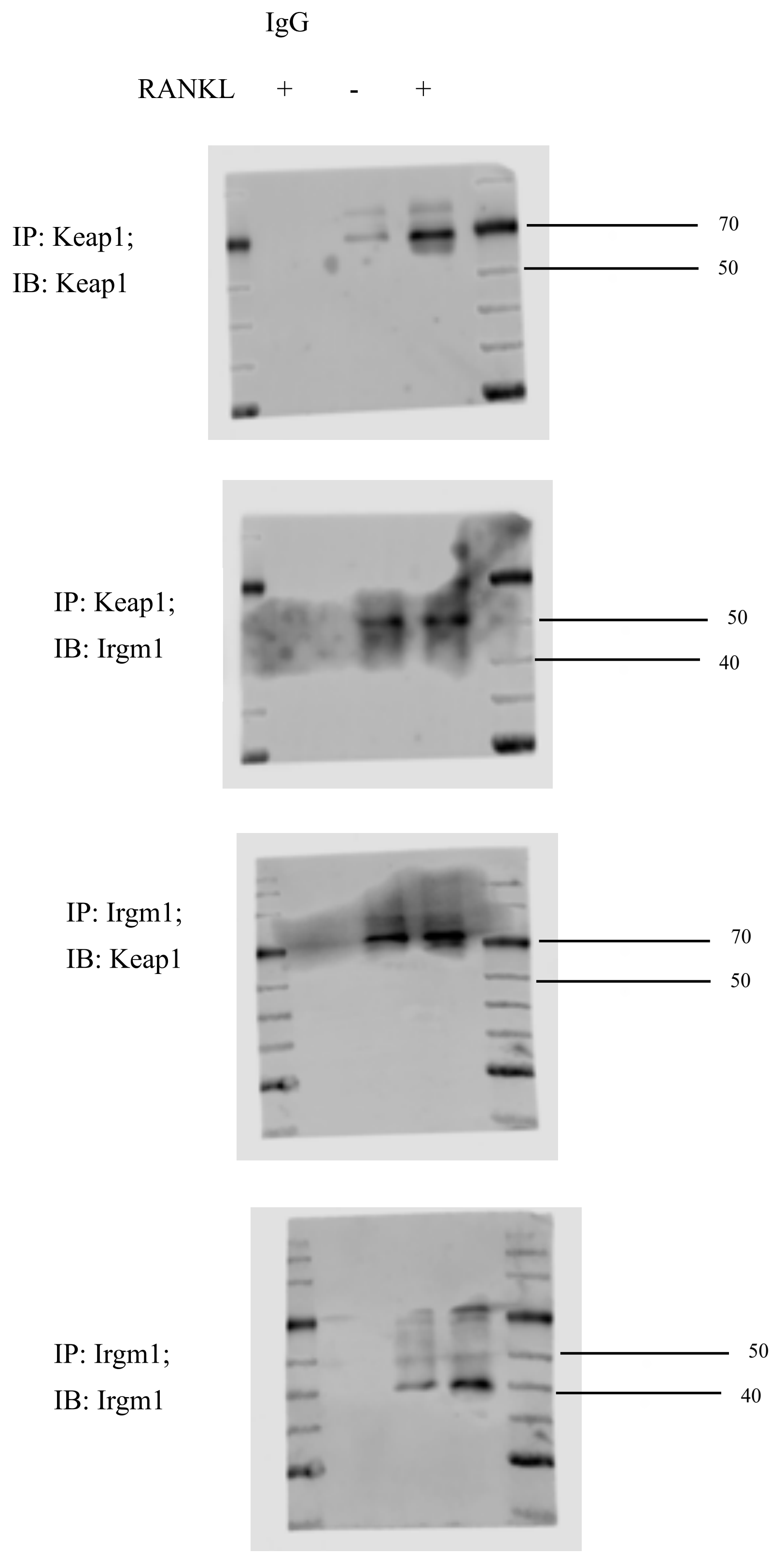
IgG
RANKL + - +
IP: Keap1;
IB: Keap1
70
50
IP: Keap1;
IB: Irgm1
50
40
IP: Irgm1;
IB: Keap1
70
50
IP: Irgm1;
IB: Irgm1
50
40

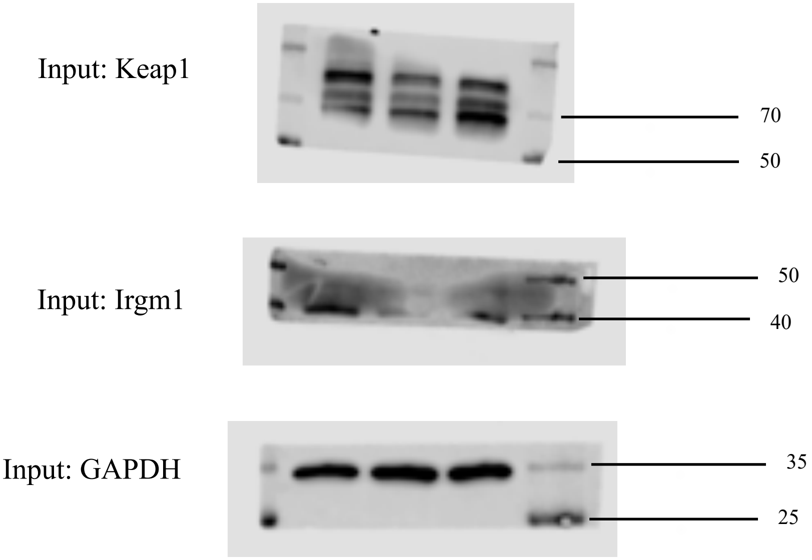
Input: Keap1
70
50
Input: Irgm1
50
40
Input: GAPDH
35
25

Figure 7E

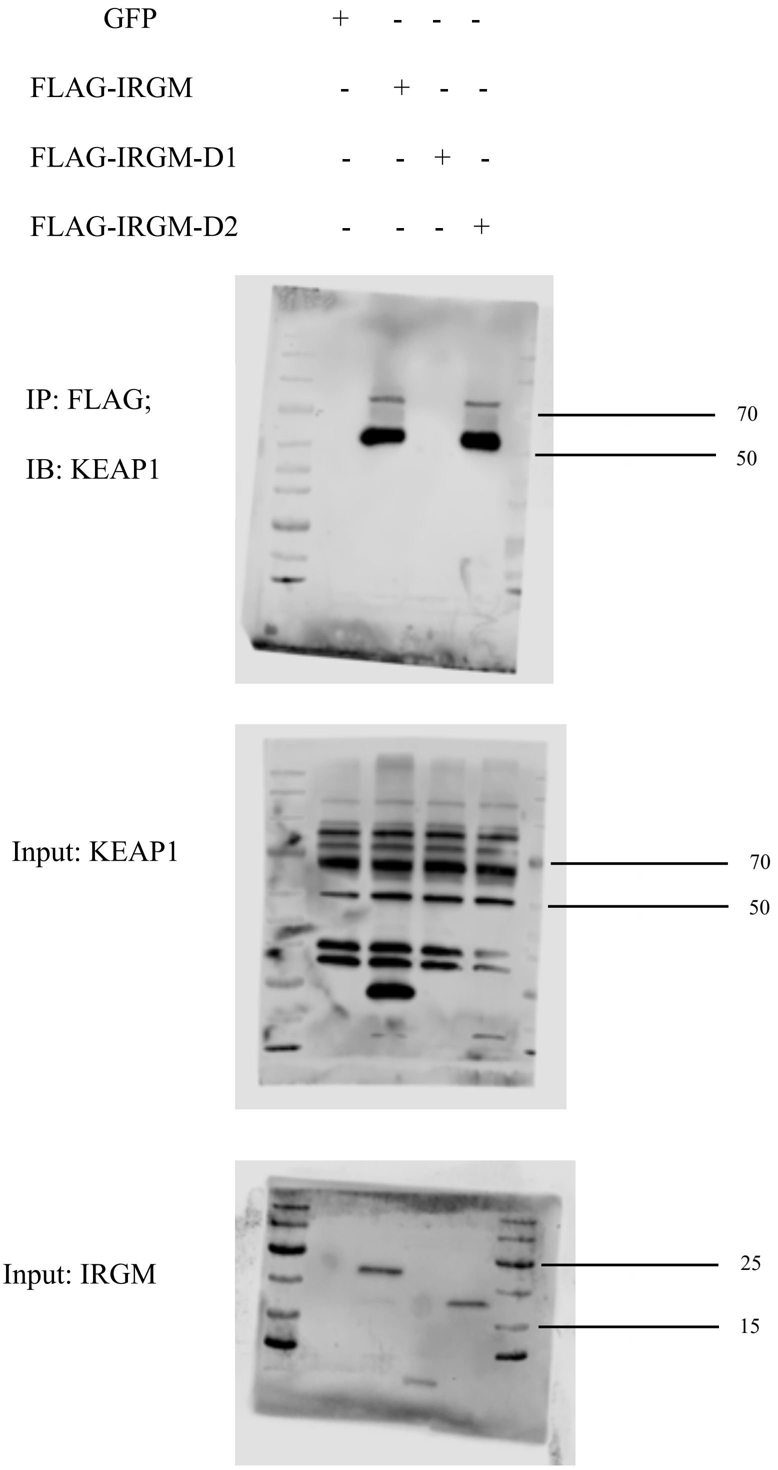
GFP + - - -
FLAG-IRGM - + - -
FLAG-IRGM-D1 - - + -
FLAG-IRGM-D2 - - - +
IP: FLAG;
IB: KEAP1
70
50
Input: KEAP1
70
50
Input: IRGM
25
15

Figure 7F

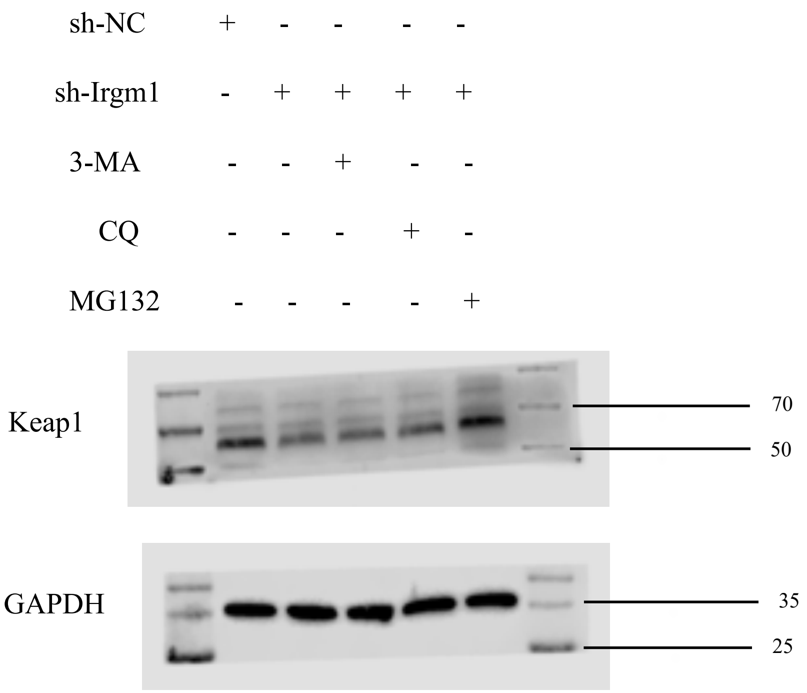

Figure 7H

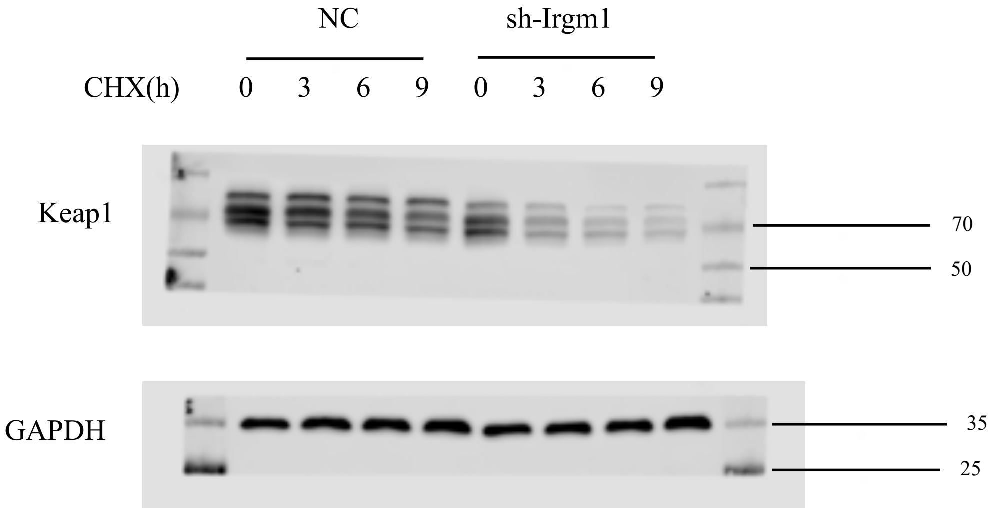

Figure 7I

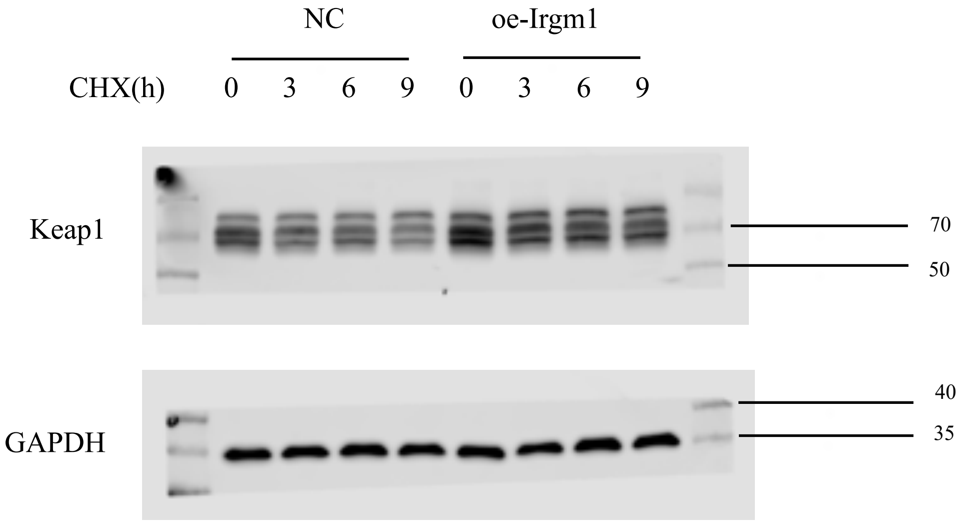

Figure 7J

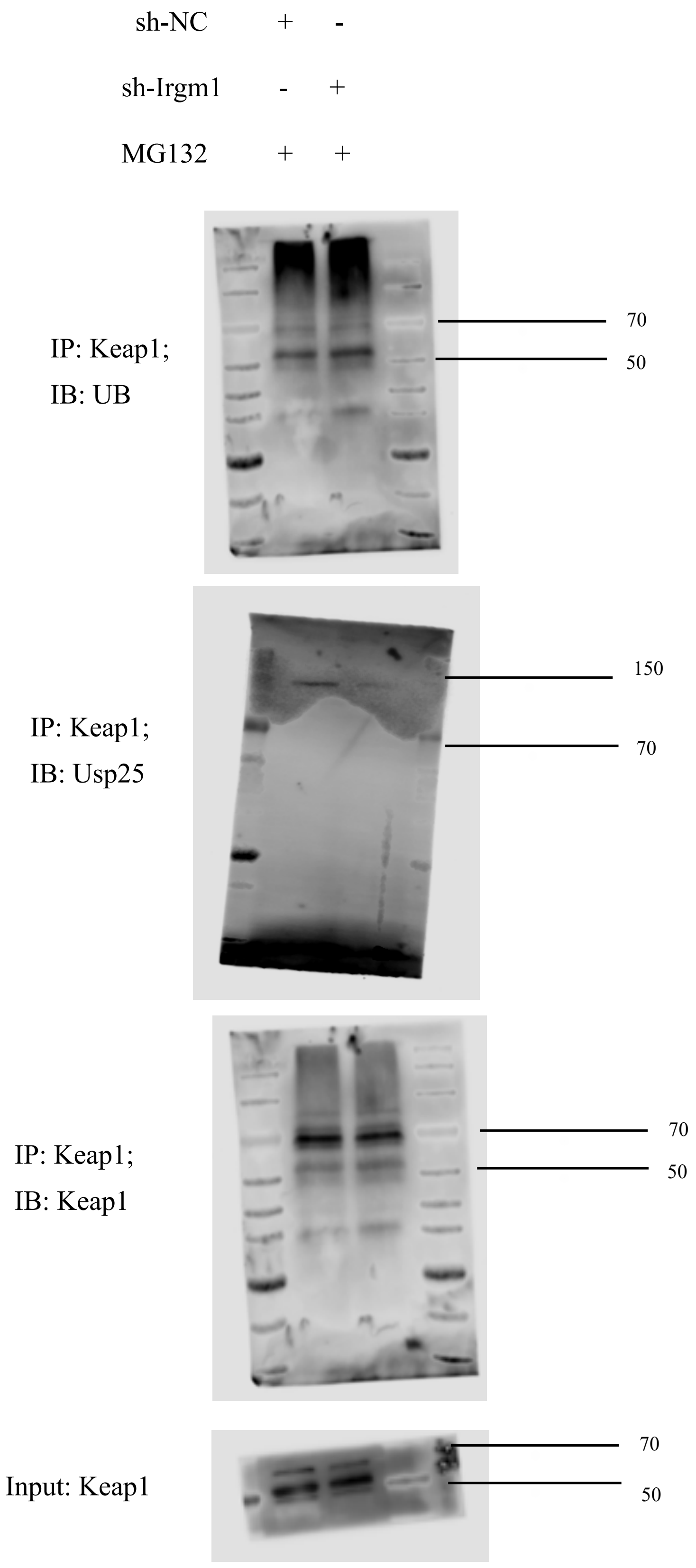

sh-NC + -
sh-Irgm1 - +
MG132 + +
IP: Keap1;
IB: UB
70
50
IP: Keap1;
IB: Usp25
150
70
IP: Keap1;
IB: Keap1
70
50
Input: Keap1
70
50

Input: Usp25

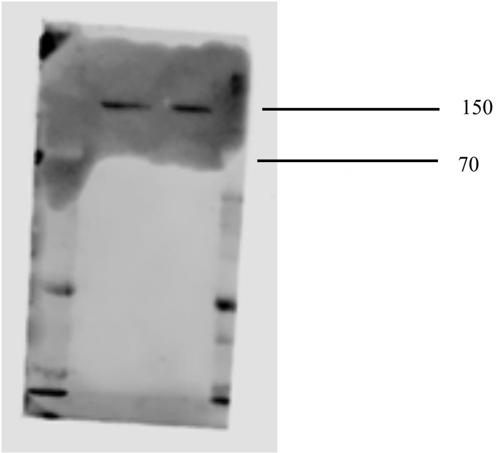

150

70

Input: GAPDH

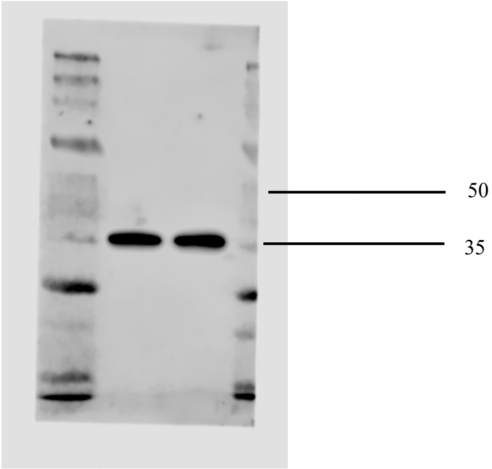

50

35

Figure 7K

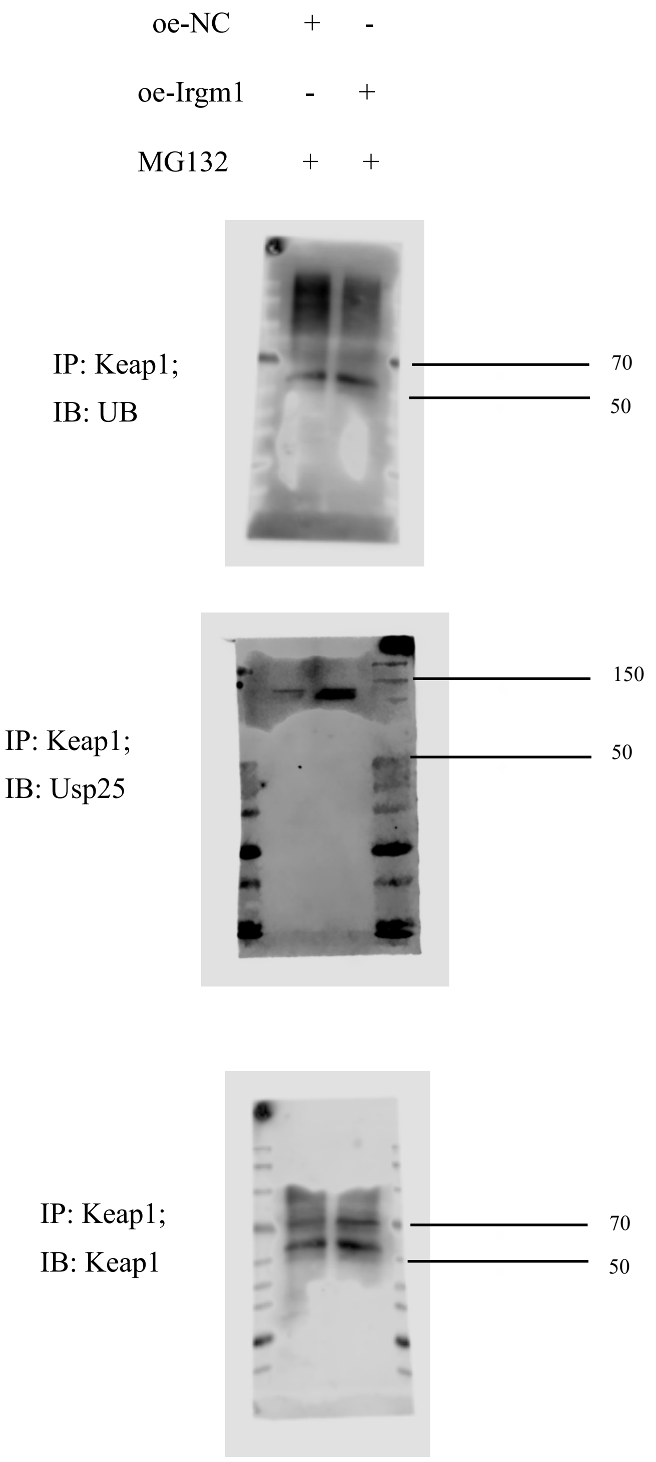
oe-NC + -
oe-Irgm1 - +
MG132 + +
IP: Keap1;
IB: UB
70
50
IP: Keap1;
IB: Usp25
150
50
IP: Keap1;
IB: Keap1
70
50

Input: Usp25

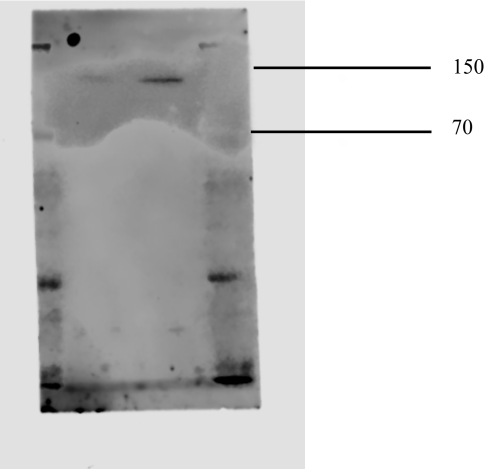

Input: Keap1

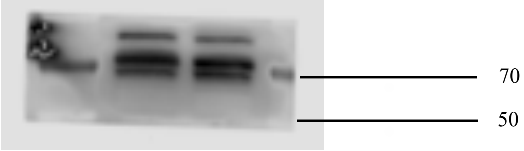

Input: GAPDH

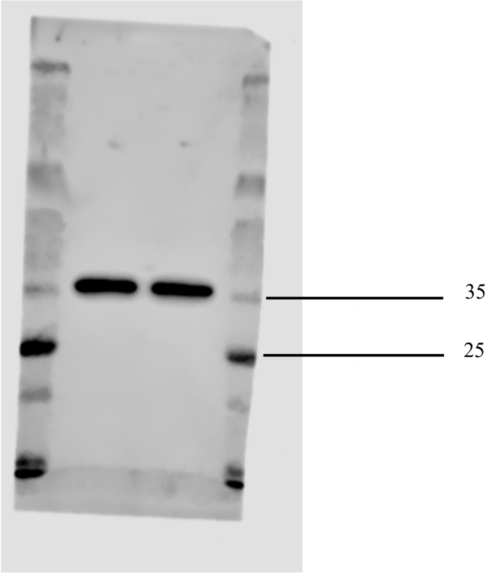

Figure 8I

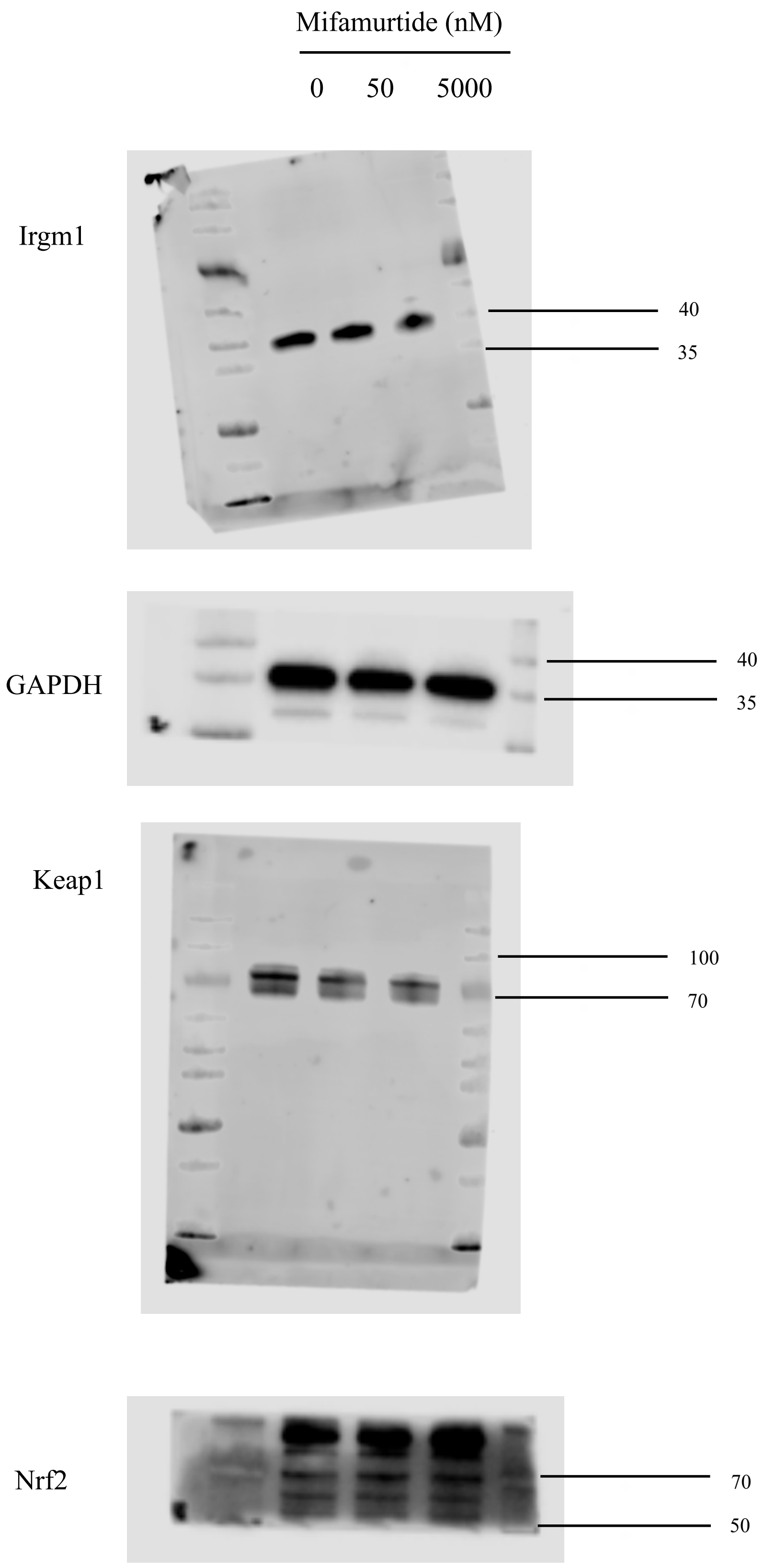
Mifamurtide (nM)
0 50 5000
Irgm1
40
35
GAPDH
40
35
Keap1
100
70
Nrf2
70
50

Figure 9E

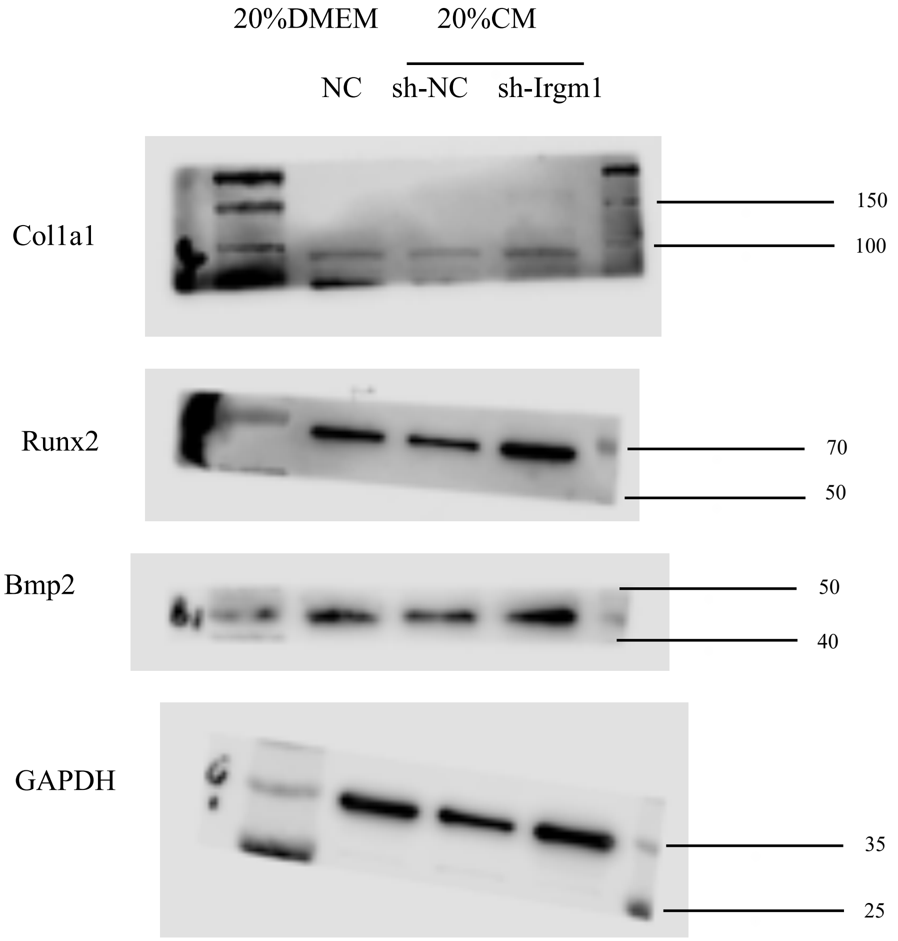
20%DMEM
20%CM
NC
sh-NC
sh-Irgm1
Col1a1
150
100
Runx2
70
50
Bmp2
50
40
GAPDH
35
25

Figure 9I

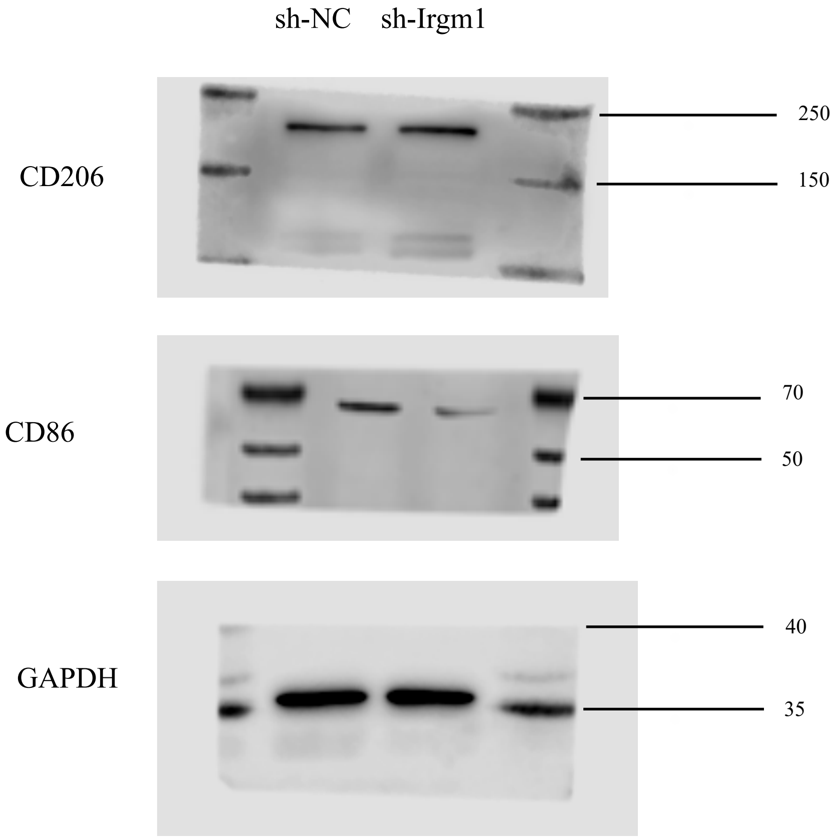

Supplement: Supplementary file 2 — Original Western blots [file 41419_2025_7965_MOESM2_ESM.pdf]
